# ﻿Towards a monophyletic *Melicope* (Rutaceae): Phylogenetic placement of *Dutailliopsis* and other New Caledonian genera, and an updated infrageneric classification of *Melicope*

**DOI:** 10.3897/phytokeys.259.139246

**Published:** 2025-07-10

**Authors:** Marc S. Appelhans, Yohan Pillon, Karine Gotty, Warren L. Wagner

**Affiliations:** 1 Integrative Plant Taxonomy and Botanical Garden, Institute for Biodiversity, Ecology and Evolution, Friedrich-Schiller University Jena, Philosophenweg 16, 07743 Jena, Germany Friedrich-Schiller University Jena Jena Germany; 2 Senckenberg Institute for Plant Form and Function (SIP) Jena, Philosophenweg 16, 07743 Jena, Germany Smithsonian Institution Washington United States of America; 3 Department of Systematics, Biodiversity and Evolution of Plants, Albrecht-von-Haller Institute of Plant Sciences, University of Goettingen, Untere Karspuele 2, 37073 Goettingen, Germany Senckenberg Institute for Plant Form and Function Jena Germany; 4 Department of Botany, National Museum of Natural History, Smithsonian Institution, PO Box 37012, Washington, DC 20013-7012, USA University of Goettingen Goettingen Germany; 5 DIADE, Université de Montpellier, CIRAD, IRD, Montpellier, France Université de Montpellier Montpellier France; 6 LSTM, IRD, INRAE, CIRAD, Institut Agro, Univ. Montpellier, Montpellier, France University Montpellier Montpellier France

**Keywords:** *Comptonella*, *Dutaillyea*, *Melicopevitiflora*, New Caledonia, *Picrella*, polyphyletic genera, *Sarcomelicope*

## Abstract

*Dutailliopsis* (Rutaceae) is a scarcely known monotypic genus from New Caledonia. It is one of the few genera of Rutaceae that has never been included in a molecular phylogenetic study. Based on Sanger sequencing of the markers ETS, ITS, and *trn*L-*trn*F, we conclude that *Dutailliopsis* is nested within the large genus *Melicope* and that it is most closely related to other New Caledonian species of *Melicope*. Previous molecular phylogenetic studies revealed that other New Caledonian genera – namely the endemic *Comptonella*, *Dutaillyea*, *Picrella*, and the near-endemic *Sarcomelicope* (only one out of the nine species is not endemic to New Caledonia) – are nested in *Melicope* as well and we propose nomenclatural changes to render *Melicope* monophyletic and develop a new classification system for the genus.

## ﻿Introduction

With about 235 species, *Melicope* J.R.Forst. & G.Forst. is the most species-rich genus of the *Citrus* family, Rutaceae (Hartley 2001; Kubitzki et al. 2011). The genus has a wide distribution ranging from the Indo-Himalayan region in the east, throughout southern, eastern and south-eastern Asia, Malesia and Australasia to most Pacific archipelagos. Additionally, *Melicope* occurs on Madagascar and the Mascarene Islands (Hartley 2001; Appelhans et al. 2018a). Molecular phylogenetic studies of *Melicope* and its close relatives *Acronychia* J.R.Forst. & G.Forst. and *Euodia* J.R.Forst. & G.Forst. revealed that a number of small genera are nested within them and that the current sectional classification of *Melicope* is not composed of monophyletic groups (Appelhans et al. 2014a, 2014b, 2018a). The genera *Comptonella* Baker.f., *Dutaillyea* Baill., *Picrella* Baill., *Platydesma* H.Mann and *Sarcomelicope* Engl. are nested within *Melicope*. *Acronychia* and *Maclurodendron* might also be nested in *Melicope*, but the statistical support for this placement is low (Appelhans et al. 2014b, 2018a). A first step towards a monophyletic circumscription of *Melicope* was the merging of the Hawaiian endemic *Platydesma* into it (Appelhans et al. 2017). The remaining genera that need to be merged into *Melicope* are either endemic to New Caledonia (*Comptonella*, *Dutaillyea*, *Picrella*), or are most diverse in New Caledonia (*Sarcomelicope*; eight of the nine species are endemic to New Caledonia) (Hartley 1982, 1983, 1984, 1986; Hartley and Mabberley 2003).

**Figure 1.**
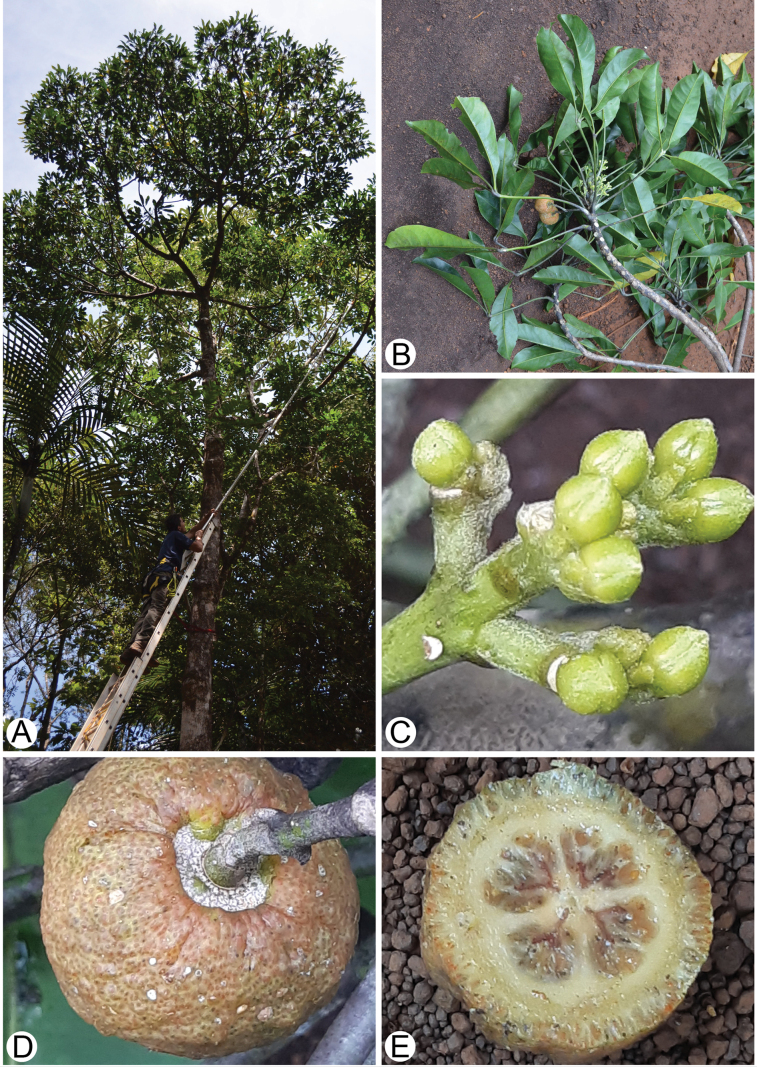
*Melicopegordonii* (T.G.Hartley) Appelhans & W.L.Wagner **A** habit **B** flowering and fruiting branch **C** partial inflorescence with flower buds **D** fruit **E** transverse section of fruit showing locules and sculptured endocarp. Photos by Ghislain Vieilledent.

The previous molecular phylogenetic studies of *Melicope* had a comprehensive taxon sampling (Appelhans et al. 2014b, 2018a), but the genus *Dutailliopsis* T.G.Hartley (Fig. 1), which is morphologically similar to *Comptonella* and *Dutaillyea*, had not been sampled. *Dutailliopsis* is sequenced here for the first time, so that only six Neotropical and monotypic genera (*Apocaulon* Cowan, *Euxylophora* Huber, *Leptothyrsa* Hook.f., *Naudinia* Planch. & Linden, *Polyaster* Hook.f., *Rutaneblina* Steyerm. & Luteyn) out of 154 genera in the family (Appelhans et al. 2021) remain to be sampled for a molecular phylogenetic study (Appelhans et al. 2021; Joyce et al. 2023). *Dutailliopsis* is a monotypic genus endemic to the south of Grande Terre (New Caledonia) that was described in 1997, based on material collected between 1983 and 1990 (Hartley 1997). It was described as a new genus especially because of the unusually sculptured endocarp, and was named *Dutailliopsis* because of its similarities to the genus *Dutaillyea*. However, lepidote or stellate trichomes, that are characteristic of *Comptonella* and *Dutaillyea*, are not present in *Dutailliopsis* (Hartley 1997).

Here, we sampled *Dutailliopsisgordonii* T.G.Hartley for sequencing for the first time. The goals of this study are (1) to ascertain the phylogenetic relationships of *Dutailliopsis* relative to *Melicope* and especially in regards to the New Caledonian species, and (2) to undertake the nomenclatural changes that are needed to establish a monophyletic *Melicope*.

## ﻿Methods

The taxon sampling of the study is largely based on Appelhans et al. (2014b, 2018a) and represents a subset of these studies with a focus on New Caledonian lineages. Only one sample of *Dutailliopsisgordonii* (*Morat 8639*, P00227775!) obtained from the herbarium P (Le Bras et al. 2017) was added, so that the final dataset consisted of 62 accessions representing all main clades of *Melicope* (Appelhans et al. 2014b, 2018a), *Acronychia*, the monotypic *Dutailliopsis*, all genera that are nested within *Melicope*, as well as *Medicosma* Hook.f. and *Tetractomia* Hook.f. as outgroups (Table 1).

**Table 1.** Voucher information, and Genbank numbers of all specimens used in this study. Herbarium acronyms are according to Index Herbariorum (http://sweetgum.nybg.org/science/ih/) and abbreviations for genera are as follows: *A. = Acronychia*, *Me. = Medicosma*, *M.* = *Melicope*, *T.* = *Tetractomia*.

Taxon	Collector & number (Herbarium)	Origin	Section	*trnL-trnF*	ITS	ETS
*A.acronychioides* (F. Muell.) T.G. Hartley	*Forster PIF30987* (L.4166710)	Australia, Queensland		LN849177	LN849136	LN849220
*A.acuminata* T.G.Hartley	*Ford 3997* (CNS)	Australia, Queensland		LN849178	LN849137	LN849221
*A.brassii* T.G.Hartley	*Appelhans 467* (US01176041)	Papua New Guinea		HG971155	HG971306	HG971460
*A.imperforata* F.Muell.	*Forster PIF30952* (L.4166712)	Australia, Queensland		LN849182	LN849143	LN849231
*A.laevis* J.R.Forst. & G.Forst.	*Forster PIF30953* (L.4166711)	Australia, Queensland		LN849183	LN849144	LN849232
*A.ledermannii* T.G.Hartley (cf)	*Appelhans 458* (US01176072)	Papua New Guinea		HG971158	HG971309	HG971463
*A.murina* Ridl.	*Regalado 1023* (A)	Papua New Guinea		LN849187	LN849146	LN849237
*A.pedunculata* (L.) Miq.	*de Wilde 6834* (L.4166578)	Thailand		HG002754	HG002398	HG002527
*A.pullei* Lauterb.	*Appelhans 460* (US01176074)	Papua New Guinea		HG971159	HG971310	HG971465
A.trifoliolataZoll. & Moritzivar.microcarpa T.G.Hartley	*Appelhans 416* (US01176051)	Papua New Guinea		HG971162	HG971313	HG971468
*M.accedens* (Blume) T.G.Hartley	*Beaman 7360* (L)	Borneo	*Lepta*	HG971173	HG971331	HG971485
*M.aneura* (Lauterb.) T.G.Hartley	*Appelhans 441* (US01176064)	Papua New Guinea	*Ptelea*	HG971177	HG971335	HG971489
*M.balgooyi* Appelhans,W.L.Wagner & K.R.Wood	*Wood 9698* (NY)	Austral Islands	*Melicope*	HG971246	HG971418	HG971571
*M.balgooyi*	*Wood 9727* (NY)	Austral Islands	*Melicope*	HG971247	HG971419	HG971572
*M.barbigera* A.Gray	*Wagner 6896* (US01154516)	Hawaii, Kaua'i	*Ptelea*	HG002793 + HG002869	HG002406	HG002535
*M.baudouinii* (Baill.) Appelhans & W.L.Wagner	*MacKee 29450* (L.4267819)	New Caledonia	*Dutaillyea*	HG971165	HG971317	HG971471
*M.bonwickii* (F.Muell.) T.G.Hartley	*Wen 10286* (US00863000)	Sulawesi	*Lepta*	HG971179	HG971337	HG971491
*M.capillacea* (Gillespie) A.C.Sm.	*Smith 4992* (NY)	Fiji	*Picrella*	HG971291	HG971342	–
*M.clusiifolia* (A.Gray) T.G.Hartley & B.C.Stone	*Wood 8151* (PTBG)	Hawaii, Kaua'i	*Ptelea*	HG002798 + HG002874	HG002412	HG002542
*M.denhamii* (Seem.) T.G.Hartley	*Appelhans 464* (US01176038)	Papua New Guinea	*Lepta*	HG971192	HG971357	HG971509
*M.durifolia* (K.Schum.) T.G.Hartley	*Appelhans 465* (US01176039)	Papua New Guinea	*Ptelea*	HG971197	HG971362	HG971514
*M.elleryana* (F.Muell.) T.G.Hartley	*Appelhans 414* (US01176018)	Papua New Guinea	*Lepta*	HG971208	HG971373	HG971525
*M.follicularis* (T.G.Hartley) Appelhans & W.L.Wagner	*Munzinger 668* (MO-265659)	New Caledonia	*Sarcomelicope*	HG971303	HG971448	HG971601
*M.glaberrima* Guillaumin	*Munzinger 927* (P00239209)	New Caledonia	*Dutailliopsis*	HG971252	HG971426	HG971579
*M.glaberrima*	*Munzinger 1111* (P00239492)	New Caledonia	*Dutailliopsis*	HG971300	HG971425	HG971578
*M.glandulosa* (T.G.Hartley) Appelhans & W.L.Wagner	*McKee 3189* (US)	New Caledonia	*Picrella*	HG971268	HG971444	HG971597
*M.glandulosa*	*McPherson 18598* (MO-325854)	New Caledonia	*Picrella*	HG971269	HG971445	HG971598
*M.gordonii* (T.G.Hartley) Appelhans & W.L.Wagner	*Morat 8639* (P00227775)	New Caledonia	*Dutailliopsis*	PP591901	PP598866	PP947748
*M.hartleyi* Appelhans & W.L.Wagner	*McPherson 18023* (MO-245279)	New Caledonia	*Dutaillyea*	HG971276 + HG971288	HG971322	HG971475
*M.hartleyi*	*Van Balgooy 7053* (L.4267823)	New Caledonia	*Dutaillyea*	HG971167	HG971323	HG971476
*M.ignambiensis* (Guillaumin) Appelhans & W.L.Wagner	*McPherson 19132* (MO-398139)	New Caledonia	*Picrella*	HG971284 + HG971302	HG971446	HG971599
*M.lasioneura* (Baill.) Baill ex. Guillaumin (cf)	*Munzinger 939* (P)	New Caledonia	*Dutailliopsis*	HG971296	HG971380	–
*M.latifolia* (DC.) T.G.Hartley	*Polak 1044* (L.2130144)	New Guinea	*Lepta*	HG002820 + HG002896	HG002440	HG002575
*M.lucida* (A.Gray) A.C.Sm.	*Meyer 808*	Tahiti	*Melicope*	HG971217	HG971384	HG971535
*M.lucida*	*Florence 11461* (US03307334)	Tahiti	*Melicope*	-	MG595168	MG668946
*M.lunu-ankenda* (Gaertn.) T.G.Hartley	*Stone 16055* (US03306693)	Malaysia	*Lepta*	HG971218	HG971385	HG971536
*M.madagascariensis* (Baker) T.G.Hartley	*Ramananjanakary 410* (MO)	Madagascar	*Lepta*	HG971219	HG971386	HG971537
*M.mantellii* Buchanan	*Pelser 3122* (GOET)	New Zealand	*Melicope*	MG668990	MG595159	MG668947
*M.mantellii*	*Gardner 670* (L.2124285)	New Zealand	*Melicope*	MG668991	MG595160	MG668948
*M.margaretae* (F.Br.) T.G.Hartley	*Meyer 1003* (NY)	Austral Islands	*Picrella*	HG971221	HG971388	HG971539
*M.margaretae*	*Perlman 17954* (NY)	Austral Islands	*Picrella*	HG971222	HG971389	HG971540
*M.microcarpa* (Perkins) Appelhans & W.L.Wagner	*Munzinger 679* (MO-267298)	New Caledonia	*Dutaillyea*	HG971274 + HG971286	HG971318	HG971472
*M.microcarpa*	*Lowry 5734* (MO-343210)	New Caledonia	*Dutaillyea*	HG971275 + HG971287	HG971319	HG971473
*M.oblanceolata* T.G.Hartley	*Appelhans 462* (US01176036)	Papua New Guinea	unplaced	–	HG971394	HG971546
*M.oreophila* (Guillaumin) Appelhans & W.L.Wagner	*McPherson 18544* (MO-325896)	New Caledonia	*Dutaillyea*	HG971166	HG971320	HG971474
*M.pachypoda* (Lauterb.) T.G.Hartley	*Appelhans 447* (US01176068)	Papua New Guinea	*Lepta*	HG971229	HG971399	HG971551
*M.polybotrya* (C.Moore & F.Muell.) T.G.Hartley	*Hutton 284* (CANB)	Lord Howe Island	*Picrella*	EU493240	EU493183	HG971554
*M.ponapensis* Lauterb.	*Tangalin 1208* (PTBG)	Caroline Islands, Pohnpei	*Ptelea*	HG002770	HG002464	HG002602
*M.pteleifolia* (Champ. ex Benth.) T.G.Hartley	*Wen 11376* (US01175641)	China	*Lepta*	HG971234	HG971404	HG971557
*M.rubra* (Lauterb. & K.Schum.) T.G.Hartley	*Appelhans 425* (US01176024)	Papua New Guinea	*Lepta*	HG971237	HG971408	HG971561
*M.simplex* A.Cunn.	*Gardner 3188* (L.4267654)	New Zealand	*Melicope*	HG002847 + HG002923	HG002486	HG002627
*M.simplex*	*Pelser 3121* (GOET)	New Zealand	*Melicope*	MG668996	MG595165	MG668954
*M.sororia* T.G.Hartley	*Appelhans 384* (US)	Borneo	unplaced	HG971245	HG971417	HG971570
*M.spathulata* A.Gray	*Wood 14213* (PTBG)	Hawaii, Kaua'i	*Ptelea*	HG002860 + HG002939	HG002508	HG002650
*M. spec.*	*Munzinger 785* (MO-268453)	New Caledonia	*Picrella*	HG971282	HG971420	HG971573
*M. spec.*	*Munzinger 790* (MO-268449)	New Caledonia	*Dutaillyea*	HG971277	HG971324	HG971477
*M.ternata* J.R.Forst. & G.Forst.	*Appelhans 487* (GOET)	Cultivated Botanical Garden Göttingen	*Melicope*	HG971258	HG971432	HG971585
*M.triphylla* (Lam.) Merr.	*Appelhans 394* (GOET)	cultivated Hortus Botanicus Leiden	*Ptelea*	HG002780	HG002489	HG002630
*M.vatiana* (Setch.) T.G.Hartley	*Whistler 4170* (US01207253)	Samoa	*Ptelea*	HG002850 + HG002926	HG002490	HG002631
*M.vieillardii*	*McPherson 18066* (MO-275707)	New Caledonia	*Dutailliopsis*	HG002781	HG002491	HG002632
*Me.glandulosa* T.G.Hartley	*Forster 25045* (L.4267623)	Australia, Queensland	Outgroup	HG971172	HG971330	HG971484
*T.tetrandrum* (Roxb.) Merr.	*Beaman 8917* (L.4266883)	Borneo	Outgroup	HG971271	HG971449	HG971602

Total DNA was extracted from the *Dutailliopsisgordonii* herbarium specimen following a modified cetyltrimethylammonium bromide (CTAB) protocol (Doyle and Doyle 1987). The nuclear ETS (external transcribed spacer) and ITS (internal transcribed spacer), and the plastid *trn*L-*trn*F regions were amplified as described in Appelhans et al. (2014b) using standard primers (Taberlet et al. 1991; Maslin 2001; Murphy et al. 2010). Cleaned PCR products were sequenced using an ABI 3730 DNA sequencer.

The new *Dutailliopsis* sequences were manually added to the alignments from Appelhans et al. (2018a) using the CLC genomics workbench version 20 (Qiagen, Aarhus, Denmark) and the alignments were trimmed to contain only the 62 accessions mentioned above (Table 1).

Phylogenetic analyses were performed using Bayesian Inference (BI; MrBayes 3.2.6; Ronquist et al. 2012) and Maximum likelihood (ML; RAxML 8.2.4.; Stamatakis 2014). jModelTest 2.1.3 (Darriba et al. 2012) was used to determine the best-fitting substitution model for the three genetic markers under the Akaike Information Criterion (AIC). As a result, the GTR+G model was used for ETS, while GTR+I+G was used for ITS and *trn*L-*trn*F.

Bayesian analyses consisted of four independent MCMC runs observed for 10 million generations and sampling every 1000^th^ generation. All runs reached stationarity (standard deviation of split frequencies < 0.01) within the 10 million generations. 50% Majority-Rule consensus trees were calculated in MrBayes after discarding the first 25% of the trees as burnin. Posterior probability (PP) values of ≥ 0.95 PP were considered as strong support for clades. RAxML analyses were based on 1000 bootstrap replicates and using the same substitution models as in the BI analyses. Bootstrap (BS) values of 50%–69% were considered to indicate low support, values of 70%–89% as moderate support, and values of ≥ 90% as strong support.

## ﻿Results

**Figure 2.**
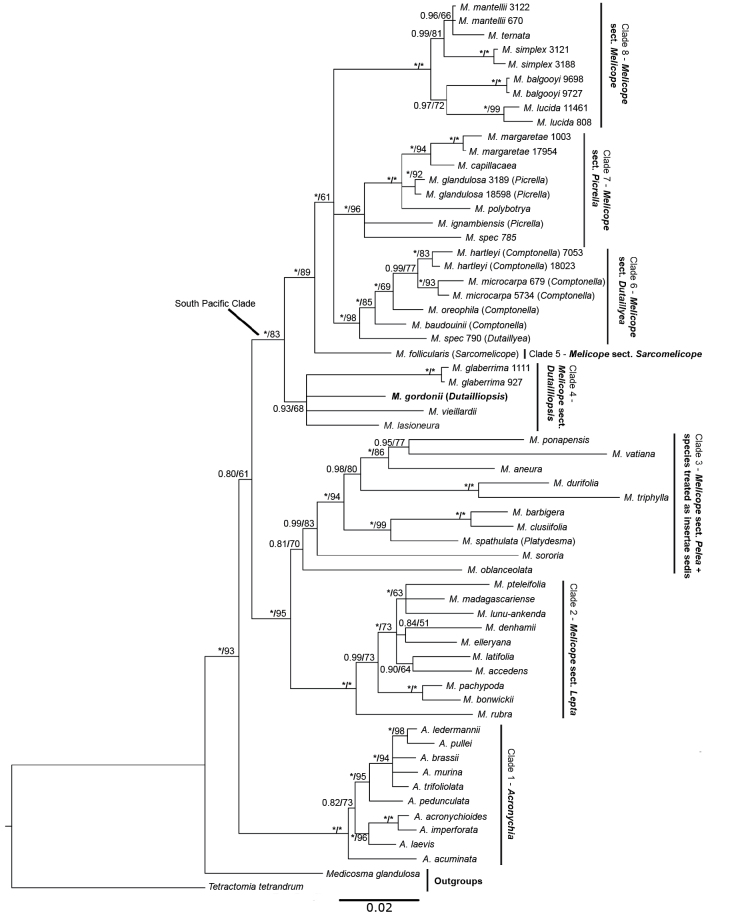
Phylogenetic reconstruction of *Acronychia* and *Melicope* based on ETS, ITS, and *trnL-trnF*. Bayesian Posterior Probabilities and ML Bootstrap Support values are shown next to the branches. An asterisk (*) marks cases of full support (1.00pp/100% bs). Collecting numbers are mentioned for specimens in case more than one sample has been included per species. Previous genus names have been added in brackets for species previously assigned to *Comptonella*, *Dutailliopsis*, *Dutaillyea*, *Picrella*, *Platydesma* and *Sarcomelicope*. Abbreviations: *A.* = *Acronychia*, *M.* = *Melicope*.

The phylogenetic trees obtained here (Fig. 2) are largely congruent to the consensus trees from the previous studies (Appelhans et al. 2014b, 2018a). The only significant difference is that the *Acronychia* clade is sister to *Melicope* with low support in the present study, while it was nested within *Melicope* with low support in previous studies (Appelhans et al. 2014b, 2018a). A more recent study based on High-Throughput Sequencing (Target Capture) also resolved *Acronychia* and *Melicopesensu lato* as sister genera, although with limited taxon sampling of these genera (Joyce et al. 2023).

Within *Melicope*, the New Caledonian species of Melicopesect.Pelea, species of Melicopesect.Vitiflorae, all species of the revised Melicopesect.Melicope (Appelhans et al. 2014c) as well as the genera *Comptonella*, *Dutailliopsis*, *Dutaillyea*, *Picrella* and *Sarcomelicope* form a clade with high support (1.00pp) in the Bayesian analyses and moderate support (83% bs) in the ML analysis (Fig. 2, South Pacific Clade). Sister to this South Pacific Clade is a species rich clade that consists of Melicopesect.Lepta (102 species; Hartley 2001; Clade 2), as well as the non-New Caledonian species of Melicopesect.Pelea (92 species; Hartley 2001; Appelhans et al. 2017; Wood et al. 2016, 2017, 2024) and the species of Melicopesect.Melicope (33 species; Hartley 2001) sensu Hartley (2001) that have been excluded from the section by Appelhans et al. (2014c) (Clade 3). The 33 species that have been excluded from Melicopesect.Melicope form a grade to Melicopesect.Pelea (Appelhans et al. 2014b, 2018a), which is exemplified by *M.oblanceolata* T.G.Hartley and *M.sororia* T.G.Hartley in Fig. 2.

The earliest diverging subclade (Clade 4) within the South Pacific clade is not well supported (0.93pp, 68% bs) and its internal relationships are unresolved. It contains all species of the New Caledonian Melicopesect.Pelea and the newly sampled *Dutailliopsis*. The next subclade (Clade 5) consists of the genus *Sarcomelicope* (1.00pp, 89% bs). Three additional subclades (Clades 6, 7, 8) include the remainder of the species. While these are supported as a whole (1.00pp, 61% bs), the relationships among the three clades were not resolved. Clade 6 consists of *Comptonella* and *Dutaillyea* (1.00pp, 98% bs), and both genera are resolved as monophyletic. Clade 7 contains the polyphyletic genus *Picrella* and all sampled species of Melicopesect.Vitiflorae (1.00pp, 96% bs) which is also polyphyletic. *Melicopevitiflora* was not included in this analysis since it was shown previously that it does not belong to *Melicope* and is more closely related to *Euodia* (Appelhans et al. 2014b, 2018a). *Picrella* is not resolved as monophyletic. The final clade (Clade 8) contains all species of Melicopesect.Melicope sensu Appelhans et al. (2014c) that are found from New Zealand to Tahiti and the Austral Islands (1.00pp, 100% bs; none of the species is found in New Caledonia).

## ﻿Discussion

### ﻿Evolution of drupaceous fruits in *Melicope* and placement of *Dutailliopsis*

For a long time, the definition of subfamilies in Rutaceae had been largely based on fruit characters (Engler 1931). Engler’s classification was used without significant revision, until morphological (Hartley 2001), phytochemical (Waterman 2007) and molecular phylogenetic studies (Poon et al. 2007; Appelhans et al. 2021) showed that – except for subfamily Aurantioideae – fruit characters are not suited to define subfamilies in Rutaceae. Several sister group relationships of genera with dehiscent and indehiscent fruits have been identified (Appelhans and Wen 2020) and a new subfamily classification has recently been proposed, which does not focus on fruit characters (Appelhans et al. 2021).

Most *Melicope* species sensu Hartley (2001) have capsular or follicular fruits, but several species have a (sub)fleshy exocarp (Hartley, 2001 p. 19). Species in the genus *Acronychia* usually have drupaceous fruits in which carpels are either basally connate or fully syncarpous. One species, *A.octandra* (F.Muell.) T.G.Hartley, which is sister to all other *Acronychia* species (Holzmeyer et al. 2015), has dry fruits with a chartaceous epicarp and a prominent suture, but the fruits do not dehisce (Hartley 1974, 1991). These intermediate fruit types indicate that the differences between follicles and capsules on the one hand and drupes on the other hand are not as distinct in Rutaceae as the terms suggest.

Within the *Melicope* clade, indehiscent and fleshy fruits only occur in the South Pacific clade (Fig. 2) and all taxa with indehiscent fruits are currently treated as independent genera: *Comptonella*, *Dutailliopsis*, *Dutaillyea*, *Picrella* and *Sarcomelicope*. Our analyses show that *Comptonella* and *Dutaillyea* are sister groups, but the other taxa are not immediate relatives, and each are more closely related to “typical” *Melicope* species than to one another (Fig. 2). Thus, drupaceous fruits are found in four different lineages of *Melicopesensu lato.* In order to delimit monophyletic genera, the above-mentioned genera need to be merged into *Melicope*, or *Melicope* would have to be split into multiple genera. Splitting *Melicope* would decrease the number of species in the genus from about 235 to only five or six, and it would result in the description of at least five genera that would be very difficult to define on a morphological basis. Merging the genera into *Melicope* requires broadening the circumscription of the genus, and specifically to add drupaceous fruits in addition to follicular and capsular fruits in the genus circumscription, which given the grade seen is not contentious. A similar situation is found in *Zanthoxylum* L., the other large genus in Rutaceae, where the monotypic *Toddalia* Juss. with drupaceous fruits was recently merged into *Zanthoxylum*, which otherwise has follicular and capsular fruits (Appelhans et al. 2018b; Reichelt et al. 2021). In this article, we opted for the merging of *Comptonella*, *Dutailliopsis*, *Dutaillyea*, *Picrella* and *Sarcomelicope* into *Melicope*, and the necessary nomenclatural changes are proposed here.

As the name suggests, *Dutailliopsis* is morphologically most similar to *Dutaillyea*, but it clearly differs from it by its sharply sculptured endocarp (Hartley 1997). The clearest morphological synapomorphy of *Dutaillyea* and *Comptonella* are their lepidote trichomes (Hartley 1983, 1984), and this character is lacking in *Dutailliopsis*. Despite the overall similarity, it is therefore not surprising that *Dutailliopsis* is not an immediate relative of *Dutaillyea*. *Dutailliopsis* is most closely related to species of the New Caledonian Melicopesect.Pelea, which is surprising from a morphological point of view. The only morphological similarity of these taxa is the infertility of the antipetalous stamens (Hartley 1997, 2001) in *Dutailliopsis* and three out of the five species of New Caledonian Melicopesect.Pelea (*M.fulva* (Guillaumin) B.C.Stone, *M.glaberrima* Guillaumin, *M.pedicellata* T.G.Hartley). Most taxa in the other clades have either eight fully developed stamens (Clades 5, 8; Hartley 1982, 1986, 2001), or only one whorl of four stamens (Clades 6, 7; Hartley 1983, 1984; Hartley and Mabberley 2003). Only two species in Clade 6 – the two species of *Dutaillyea* – also have four stamens plus four antipetalous staminodes (Hartley 1984).

### ﻿Biogeography of New Caledonian *Melicope*

All New Caledonian species of *Melicope* are part of the South Pacific Clade (Fig. 2, Clades 4, 5, 6, 7, 8). Together with the species of *Comptonella*, *Dutailliopsis*, *Dutaillyea*, *Picrella* and *Sarcomelicope*, the majority of the species in the clade (27 out of 41) are endemic to New Caledonia, and one species occurs from Australia to Fiji, including New Caledonia. Within the South Pacific Clade, Clades 4 and 6 are endemic to New Caledonia. All species in Clade 5 occur in New Caledonia and only one extends beyond New Caledonia (from Australia to Fiji). Clade 7 contains three New Caledonian species and the other species are distributed from Norfolk Island, Lord Howe Island and Vanuatu to the Cook, Society and Austral Islands. Clade 8 is absent from New Caledonia and its species range from New Zealand, Kermadec Island to the Society and Austral Islands (Hartley 1982, 1983, 1984, 1986, 1997, 2001; Hartley and Mabberley 2003; Appelhans et al. 2014c).

Although the backbone phylogeny is only strongly supported in the Bayesian analyses and some nodes could not be resolved, the most parsimonious scenario is a single colonization event to New Caledonia (represented by the South Pacific Clade), with multiple subsequent dispersals to neighbouring Pacific islands. It is a scenario that has been observed in several other groups including *Plerandra* A.Gray (Araliaceae; Plunkett and Lowry 2012), *Oxera* Labill. (Lamiaceae; Barrabé et al. 2015), and palms (Arecaceae; Pérez-Calle et al. 2024). The appearance of indehiscent fruits seems concomitant with the colonisation of New Caledonia, and the reversal to dehiscent fruits often match dispersal out of New Caledonia. Nothing is known about the seed dispersers of the drupaceous New Caledonian taxa in this clade, but the capsular/follicular fruited species of *Melicope* have been associated with bird-dispersal (Hartley 2001; Appelhans et al. 2018a). Birds are attracted by the shiny, black seeds that remain attached to the open fruit, and the nutritious sarcotesta (Hartley 2001; Appelhans et al. 2018a). Birds could thus have played a crucial role in dispersing the dehiscent-fruited species to nearby islands systems in the South Pacific.

Together with New Zealand, New Caledonia is part of the Zealandia tectonic plate, and there is evidence that New Caledonia was completely or nearly completely submerged during Paleocene until 25–34 Ma (Maurizot and Campbell 2020). Molecular dating suggests that the South Pacific Clade started to diversify in the Mid to Late Miocene (mean age estimate: 10.0 mya; 95% HPD: 6.1 to 15.1 mya), which fits with a colonization of a re-emerged New Caledonian landmass (Appelhans et al. 2018a).

### ﻿Morphological characters and definition of sections

Based on this study and previous phylogenetic analyses (Appelhans et al. 2014b, 2018a), the genera *Comptonella*, *Dutailliopsis*, *Dutaillyea*, *Picrella* and *Sarcomelicope* need to be included in an enlarged genus *Melicope* and the circumscription of its sections need to be revised.

In the latest monograph of the genus, Hartley (2001) divided *Melicope* into four sections: *Lepta*, *Melicope*, *Pelea* and *Vitiflorae*. Of these, only the most species rich sect. Lepta proved to be monophyletic (Appelhans et al. 2014b, 2018a) and no taxonomic changes are needed for this section. Section Pelea would only be monophyletic if the species from New Caledonia were excluded from it (Appelhans et al. 2014b, 2018a). Hartley (2001) included the five New Caledonian species in sect. Pelea, but noted, that they have “no obvious close relatives” in the section and “are probably relicts” (Hartley, 2001 p. 31), and he lists morphological characters occurring in one or several New Caledonian species that are unusual for sect. Pelea. In agreement with our phylogenetic reconstruction, they are here regarded as part of section Dutailliopsis. Section Melicope consists of a core group of five or six species distributed in New Zealand and the South Pacific, but absent from New Caledonia. The other species of sect. Melicope (distributed from India to Australia) formed a grade to sect. Pelea in previous analyses (Appelhans et al. 2014b, 2018a; grade represented by *M.oblanceolata* and *M.sororia* in Fig. 2), but the taxon sampling in that grade is not sufficient to draw conclusions as to whether they should be merged into sect. Pelea or whether new sections should be defined for them. Section Melicope as treated here consists of the core group from New Zealand and the South Pacific, while the species that are more closely related to sect. Pelea are treated as insertae sedis here. Section Vitiflorae has also been shown to be polyphyletic, with *M.vitiflora*, the type species, being closely related to *Euodia*, while the other species are related to *Picrella* from New Caledonia (Clade 7 in Fig. 2; Appelhans et al. 2014b, 2018a).

Here, we propose *Melicope* be classified into 7 sections. Five of these are recircumscribed to accommodate the former genera *Comptonella*, *Dutailliopsis*, *Dutaillyea*, *Picrella* and *Sarcomelicope* as well as the core group of Melicopesect.Melicope, the New Caledonian species of Melicopesect.Pelea and all species of Melicopesect.Vitiflorae except *M.vitiflora* (Table 2). The seven sections and the genus as a whole are monophyletic. The number of species in *Melicope* has increased from about 235 to about 260 and the number of New Caledonian species of *Melicope* increased from five to 28.

**Table 2.** Distributional range and morphological characters of the five (re)defined sections of *Melicope*.

	Sect. Dutailliopsis	Sect. Dutaillyea	Sect. Lepta	Sect. Melicope	Sect. Pelea	Sect. Picrella	Sect. Sarcomelicope
**Distribution**	New Caledonia	New Caledonia	India to the Japanese Bonin Islands, throughout Malesia and the South Pacific until Tonga; also on Madagascar and the Mascarene Islands	New Zealand, South Pacific	New Guinea throughout most Pacific Archipelagos until the Hawaiian Islands and the Marquesas Islands; one widespread species [M.triphylla] also found further westward until Borneo, the Philippines, Taiwan and the Japanese Ryukyu Islands	Widespread in the Pacific (New Caledonia and Lord Howe Island to Society and Austral Islands)	New Caledonia, one species from Australia to Fiji
**Phyllotaxis**	opposite (rarely subopposite or in whorls of three)	opposite	opposite	opposite (rarely alternate to subopposite)	opposite or whorls of three or four	opposite	opposite or whorls of three or four
**Leaflet arrangement**	trifoliolate, unifoliolate	trifoliolate, unifoliolate	trifoliolate, unifoliolate	trifoliolate, unifoliolate	trifoliolate, unifoliolate	trifoliolate, unifoliolate	unifoliolate
**Flower reproductive morphology**	dioecious, rarely bisexual or andromonoeceous	dioecious (8 spp.), bisexual (2 spp.)	dioecious or bisexual	dioecious or bisexual	dioecious or bisexual	dioecious, rarely bisexual or polygamous	dioecious
**Number of stamens**	8, or 4 + 4 staminodes	4 or 4 + 4 staminodes	4	8	8 (4 to 8 in 2 spp.)	4 (8 in 1 spp.)	8
**Indumentum of staminal filaments**	variable (glabrous, pubescent, hirsutulous, pilose, villous)	sparsely to densely pubescent from middle to base, rarely ciliate toward base and pilose adaxially at about the middle, or glabrous	glabrous in most species; few species sparely pubescent, or pubescent-villous to tomentose	glabrous, rarely sparsely strigulose in proximal 1/5	glabrous in most species; few species sparely pubescent, hirsutulous, pilosulose, ciliate or villous	glabrous, rarely pilosulose	ciliate
**Carpel connation**	basally connate (1 spp. syncarpous)	basally connate to syncarpous	basally connate to syncarpous	basally connate	basally connate to syncarpous	apocarpous, basally connate or rarely connate up to 1/4 the carpel length	basally connate to syncarpous
**Ovules per locule**	2	2	2	2 (unknown for one species)	2	1 or rarely 2	2
**Fruit type**	follicular (5 spp.), drupaceous (1 spp.)	drupaceous	Follicular or capsular	follicular	Follicular or capsular	follicular (7 spp.), drupaceous (3 spp.)	drupaceous
**Other particular characters**	often sepals and/or petals persistent and sometimes accrescent; endocarp strongly sculptured (serrate-winged) in 1 spp.	stellate to lepidote trichomes (1 spp. glabrous)		leave bases in two spp. strongly revolute and auriculate	staminal filaments connate in the spp. of the former genus *Platydesma*; leaves exclusively unifoliolate in the 55 Hawaiian spp.		petiole apically swollen; sepals petals and stamens persistent or subpersistent in fruit

Melicopesect.Dutailliopsis (Baill.) Appelhans & W.L.Wagner (Clade 4; six species, 4 sampled) is endemic to New Caledonia and is quite variable morphologically and the only character that unites a larger part of the species (four out of six; *D.gordonii*, *M.fulva*, *M.glaberrima*, *M.pedicellata*) is the four stamens alternating with antipetalous staminodes (Hartley 1997, 2001). Also, four out of six species (*M.fulva*, *M.glaberrima*, *M.pedicellata*, *M.vieillardii* (Baill.) Baill. ex Guillaumin) are characterized by persistent and accrescent sepals and petals (Hartley 2001), but these character states do not occur in the original circumscription of the genus *Dutailliopsis* (Hartley 1997). Except for the species *Melicopelasioneura* (Baill.) Baill ex. Guillaumin, the section is characterized by the possession of two unusual characters. *Melicopelasioneura* is included here despite lacking these morphological characters because of the phylogenetic evidence.

Only a single specimen/species of Melicopesect.Sarcomelicope (Baill.) Appelhans & W.L.Wagner (Clade 5), out of 9 species, was sampled in this study. Still, morphological features of this section are clear, and the group is easily defined. The section is characterized by unifoliolate leaves, eight stamens with ciliate filaments, apically swollen petioles, persistent or sub-persistent sepals, petals and stamens, drupaceous fruits, and leaves with a prominent and usually finely reticulate venation that resembles that of many *Acronychia* species (Hartley 1982, 1986; Kubitzki et al. 2011). Eight of the nine species are endemic to New Caledonia and one species is widespread, ranging from Eastern Australia to Fiji (Hartley 1982, 1986). The species that we include in this section used to be placed in two different genera: *Bauerella* Borzi and *Sarcomelicope* until 1982 (Hartley 1982). The only character separating the two is the degree of carpel connation (syncarpous in *Bauerella* vs. basally connate in *Sarcomelicope*), but the discovery of additional species revealed that this character grades from basally connate to fully syncarpous (Hartley 1982, 1986), as it is also the case for *Acronychia* and *Melicope* (Hartley 1974, 2001), as well as other Rutaceae genera (Kubitzki et al. 2011).

Melicopesect.Dutaillyea (Baill.) Appelhans & W.L.Wagner (Clade 6) consists of the genera *Comptonella* and Dutaillyea. The ten species in this section are endemic to New Caledonia and characterised by drupaceous fruits, four functional stamens (plus four staminodes in the two species originally placed in the genus *Dutaillyea*) and, for all but one species, an indumentum of stellate to lepidote trichomes ([Bibr B21][Bibr B22]). *Melicopehomedeboense* Appelhans & W.L.Wagner (=*Comptonellaglabra* T.G.Hartley) is glabrous throughout (Hartley 1983).

Like sect. Dutailliopsis, Melicopesect.Picrella (Baill.) Appelhans & W.L.Wagner (Clade 7) is composed of species with drupaceous and follicular/capsular fruits. Despite the different fruit types, the species of this section share clear similarities. The carpels in all species are apocarpous or connate only at the base and they usually contain a single locule. In two species – *M.glandulosa* (T.G.Hartley) Appelhans & W.L.Wagner (=*Picrellaglandulosa* T.G.Hartley) and *M.polybotrya* (C.Moore & F.Muell.) T.G.Hartley – the ovaries are rarely 2-loculate, and in one species – *M.bracteata* (Nadeaud) S.L. Welsh – they are generally 2-loculate (Hartley 2001; Hartley and Mabberley 2003). This latter species could not be sampled in our study, so it is not fully clear if it belongs to this section, but apart from the number of locules, it is morphologically close to other species in this section (Hartley 2001). Unilocular carpels are a very rare character in Melicopeapart fromsect.Picrella, and can otherwise only be found in *M.novoguineensis* Valeton of section Melicope sensu Hartley (Hartley 2001). With the exception of *M.glandulosa* (*Picrellaglandulosa*), which has eight stamens, all species have four stamens (Hartley 2001; Hartley and Mabberley 2003). The species in this section were formally placed in Melicopesect.Vitiflorae or in the genus *Picrella* and the distribution range of the section ranges from Vanuatu, New Caledonia, Norfolk Island and Lord Howe Island to the Cook, Society and Austral Islands (Hartley 2001; Hartley and Mabberley 2003).

Melicopesect.Melicope (Clade 9) consists of five or six species that are distributed across the South Pacific from New Zealand to Tahiti (not found in New Caledonia) and the Austral Islands (Appelhans et al. 2014c). One of the three New Zealand species, *Melicopemantellii* Buchanan, is likely a hybrid of the other New Zealand species *M.simplex* A.Cunn. and *M.ternata* J.R.Forst. & G.Forst. (Cheeseman 1906; Cockayne and Allan 1934; Fujita 1961). This section is characterized by eight stamens with glabrous filaments, carpels connate at base (fruit unknown in *M.balgooyi* Appelhans, W.L.Wagner & K.R.Wood), and some but not all species have beaked carpels and/or revolute and auriculate leaf bases (Hartley 2001; Appelhans et al. 2014c). Beaked fruits also occur in sect. Picrella, but only in combination with four stamens (Hartley 2001).

### ﻿Taxonomy

*Melicope* species are shrubs or trees (small to medium sizes, more rarely tall trees) with opposite (rarely whorled or subopposite) and uni- or trifoliolate leaves. Flowers are 4-merous, haplo- or diplostemonous. The four carpels contain 1 or 2 ovules each (5–8 in the Hawaiian species formerly recognized as *Platydesma*) and may be fully connate or connate at the base only. Fruits are either drupaceous or dehiscent with the endocarp and seeds remaining attached to the open fruit (Hartley 2001; Kubitzki et al. 2011; Appelhans et al. 2017).

Other genera with opposite leaves and 4-merous flowers include *Acronychia*, *Boronia* Sm., *Brombya* F.Muell., *Cyanothamnus* Lindl., *Euodia*, *Maclurodendron*, *Medicosma*, *Neobyrnesia* J.A.Armstr., *Perryodendron* T.G.Hartley, *Pitaviaster*, *Tetractomia*, and *Zieria* Sm. (Kubitzki et al. 2011). Phylogenetic analyses revealed that this group of genera forms a clade, with *Melicope* being most closely related to *Acronychia*, *Maclurodendron*, *Medicosma*, and *Tetractomia* (Appelhans et al. 2021; Joyce et al. 2023).

*Melicope* can easily be distinguished from *Cyanothamnus*, *Neobyrnesia*, and *Zieria*, which are mostly small-leaved subshrubs, shrubs or small trees and have linear cotyledons (Kubitzki et al. 2011). These three genera plus *Boronia*, *Brombya*, *Euodia*, and *Medicosma* share dehiscent fruits, in which seeds are elastically discharged when the fruit opens, instead of seeds remaining attached to the open fruit in dehiscent fruited *Melicope* species (Kubitzki et al. 2011). *Perryodendron* and *Tetractomia* share the dehiscent fruits with seeds remaining attached to the open fruit with dehiscent fruited *Melicope* species. *Tetractomia* clearly differs from *Melicope* by its characteristic winged seeds (Hartley 1979). *Perryodendron* has a brittle and thin testa (like its relatives *Brombya*, *Euodia*, and *Pitaviaster*), which is clearly distinct from the seeds of *Melicope* species characterized by a shiny black pellicle, thick sclerotesta and spongy sarcotesta (Hartley 2001; Kubitzki et al. 2011). In addition to some *Melicope* species, drupes are characteristic for *Acronychia*, *Maclurodendron*, and *Pitaviaster*. Drupes of *Pitaviaster* are structured differently from those of the other taxa, and they consist of a single carpel (3 of 4 carpels abortive and caducous) and have a woody mesocarp and cartilaginous endocarp instead of a woody endocarp as in the other taxa (Kubitzki et al. 2011). *Acronychia* and *Maclurodendron* are very closely related to *Melicope* and might be congeneric with *Melicope* (Appelhans et al. 2014b, 2018a). *Acronychia* and *Maclurodendron* are particularly similar to Melicopesect.Sarcomelicope, but differ from it by their imbricate sepals and deciduous petals (vs. valvate sepals and persistent petals in Melicopesect.Sarcomelicope; Hartley 1974, 1982).

In the following, we provide a modified circumscription of *Melicope* (modified from Kubitzki et al. 2011) and a key to the sections:

*Melicope* J.R. Forst. & G. Forst.

*Euodia* J.R.Forst. & G.Forst., *pro maj. parte*, Char. Gen. Pl.: 7. 1775, ed. 2: 13. 1776.

*Entoganum* Banks ex Gaertn., Fruct. Sem. Pl.: i. 331. t. 68. 1788.

*Astorganthus* Endl. ex Hook., Cat. Hort. Vindob. 2: 196. 1843.

*Pelea* A.Gray, Proc. Amer. Acad. Arts 3: 50. 1853.

*Platydesma* H.Mann, Proc. Boston Soc. Nat. Hist. 10: 317. 1866.

*Picrella* Baill., Adansonia 10: 150. 1871.

*Zieridium* Baill., Adansonia 10: 303. 1872.

*Dutaillyea* Baill., Adansonia 10: 327. 1872.

*Boninia* Planch., Ann. Sci. Nat., Bot., sér. 5, 14: 309. 1871.

*Sarcomelicope* Engl., in Engler & Prantl, Nat. Pflanzenfamilien III, 4: 122. 1896.

*Bauerella* Borzi, Bol. Orto Bot. Palermo 1: 155. 1897.

*Comptonella* Baker f., J. Linn. Soc., Bot. 45: 281. 1921.

*Tractocopevodia* Raizada & V.Naray., Indian Forester 72: 275. 1946.

*Evodiella* B.L.Linden, Nova Guinea, n.s., 10: 145. 1959.

*Dutailliopsis* T.G.Hartley, Adansonia III, 19: 210. 1997.

Shrubs or trees; often dioecious; trichomes simple, or fasciculate, stellate, sublepidote or lepidote, rarely plants completely glabrous. Leaves opposite or sometimes subopposite or whorled, digitately 3-foliolate or 1-foliolate. Inflorescences axillary to cauligerous panicles, often reduced to few or solitary flowers. Flowers bisexual or functionally unisexual, 4-merous; petals apically hooked; stamens 4 or 8 or rarely 4–8, one whirl sometimes reduced to staminodes; anthers rounded, obtuse, or mucronate at apex; stamen filaments usually separate but sometimes connate into a wide tube bearing the apically free anthers; disk pulvinate to annular, cupular, or plane; ovarioles grading from proximally to completely connate, joined (sub)apically in a common style; stylodia sometimes separating as the fruit matures; ovules 2 or 1, rarely 5–8 per carpel. Fruit in most species dehiscent and consisting of 1–4 basally connate follicles or grading to completely syncarpous (the carpels united into a 4-loculed, loculicidal capsule), or a drupe that consist of 1–4 basally connate drupelets or grading to complete syncarpous to form a 4-loculed drupe; endocarp cartilaginous, adnate or separate, but neither it nor seed discharged when fruit dehisces. Seeds 1 or 2; testa with thick sclerotesta, sarcotesta, and shiny, black pellicle; endosperm copious; cotyledons +/- flattened, elliptic.

### ﻿Key to sections of *Melicope*

**Table:** 

1a	Fruit drupaceous	**2**
2a	Indumentum of stellate or lepidote trichomes or plant glabrous throughout (*M.homedeboense*)	***sect. Dutaillyea***
2b	Indumentum of simple trichomes	3
3a	Leaf(let) venation usually prominent and finely to coarsely reticulate, staminal filaments ciliate, petiole apically swollen, sepals, petals and stamens persistent or subpersistent in fruit	***sect. Sarcomelicope***
3b	Plant without this combination of characters	**4**
4a	Flowers hermaphroditic, carpels fully syncarpous, carpels 2-loculate	***M.gordonii* (sect. Dutailliopsis)**
4b	Flowers unisexual (plants dioecious, monoecious or polygamous), carpels apocarpous or shortly connate at base, carpels 1-loculate (rarely 2-loculate in *M.glandulosa*)	***M.glandulosa* , *M.ignambiensis* , *M.trifoliata* (sect. Picrella)**
1b	Fruit dehiscent, carpels ranging from basally to fully connate	**5**
5a	Stamens 4 [but see combination of characters of *M.durifolia* in second part of this couplet]	**6**
6a	Endocarp separate from epicarp in open fruits [adnate at apex in *M.bracteata* and *M.polybotrya*; fruits unknown for *M.margaretae*], usually 1 ovule per locule [2 in *M.bracteata*, 1(2) in *M.polybotrya*], seed attachment of Type A [see Hartley 2001], carpels apocarpous or connate at base	**Dehiscent-fruited species of sect. Picrella**
6b	Endocarp adnate to epicarp in open fruits, 2 ovules per locule, seed attachment of Type B [see Hartley 2001], carpels apocarpous or grading to fully syncarpous	**sect. Lepta**
5b	Stamens 8 or 4 stamens plus 4 staminoids; rarely individual flowers with 4 stamens in New Guinean specimens of *M.triphylla*, or stamens 4 to 8 [*M.durifolia*] and then flowers unisexual with capitate functional stigma, glabrous petals 1.5–3 mm long, apically subulate filaments in fertile stamens, and 0.8–1.2 mm-long fertile gynoecium	**7**
7a	Leaves opposite or rarely subopposite [*M.balgooyi*]; Fruiting carpels connate at the base only; fruiting carpels usually with a recurved or straight beak, or, if beak absent [*M.lucida*, *M.tahitensis*], leaves with a +/- auriculate basal margin and the blade strongly revolute thereby forming domatia	**sect. Melicope**
7b	Leaves opposite or whorled; Fruiting carpels connate at their base or grading to fully syncarpous; fruiting carpels not beaked	**8**
8a	Plants exhibiting one or more of the following characters: leaf(let) margin lobed or sinuate, sepals and petals persistent in fruit, sepals and/or petals accrescent in fruit, consistently infertile antipetalous stamens; endemic to New Caledonia	**Dehiscent-fruited species of sect. Dutailliopsis**
8b	Plants not showing any of the four characteristics; not found in New Caledonia	**sect. Pelea**

#### ﻿Melicopesect.Dutailliopsis (T.G.Hartley) Appelhans & W.L.Wagner, stat. et comb. nov.

Melicopesect.Pelea (A.Gray) Hook. f. *pro Parte*, in Bentham Hooker, Gen. pl. 1: 296. 1862.

*Dutailliopsis* T.G.Hartley, Adansonia III, 19: 210. 1997.

**Type species**. *Melicopegordonii* (T.G.Hartley) Appelhans & W.L.Wagner, comb. nov.

**Note.** Six species; endemic to New Caledonia.

##### ﻿1. Melicope fulva

Taxon classificationPlantaeSapindalesRutaceae

(Guillaumin) B.C.Stone, Adansonia, nov. sér. 1: 95, tab. 1. 1961.

393EF5E0-4C99-5288-A272-14D98CEACE06

Pelea fulva Guillaumin, Bull. Soc. Bot. France 85: 302. 1938. Euodiawagapensis Guillaumin, Bull. Mus. Hist. Nat. (Paris), sér. 2, 4: 288. 1942. Type: New Caledonia, Montagnes de Wagap, 1861–1867, *Vieillard 2463 p.p.* (lectotype, designated by Hartley 2001, pg. 140: MEL [MEL68316!]; isolectotypes G [G00096078]!, GH [GH00105528!], L [L0043048!, L0043049!], NY [NY00803824!, NY00803825!], P [P00227567!], W [W0325221!]); New Caledonia, In sylvis montium [illegible] Wagap, 1861–1867, *Vieillard 2241* (syntype P [P00227780!]). Note: Another two Vieillard specimens (P00228316!, P00228318!) bear both collection numbers. They represent isolectotypes or syntypes as well, but it is unclear to which gathering they belong or if their two fragments on the sheets represent both gatherings.

###### Type material.

**New Caledonia**: Mt. Arago, 27 Nov. 1869, *Balansa 1797* (holotype P [P00543957!]; isotypes A [A00105701!], P [P00543956!, P00543958!]).

##### ﻿2. Melicope glaberrima

Taxon classificationPlantaeSapindalesRutaceae

Guillaumin, Bull. Soc. Bot. France 85: 301. 1938, as “ Melicope ?”.

0990A591-2942-56EE-AB8F-48B11B7B62ED

Pelea inotricha Guillaumin, Mém. Mus. Nati. Hist. Nat., sér. Β, Bot. 8: 69. 1957. Type: New Caledonia, Diahot sup., 31 Aug. 1951, *Hürlimann 1898* (holotype P [P00543954!]; isotypes G [G00096079!], Z [Z-000023352!, Z-000025224!]).

###### Type material.

**New Caledonia**: Forêts situées au ΝE de la Conception, 7 Jan. 1869, *Balansa 1017* (holotype P [P00543955!]).

##### ﻿3. Melicope gordonii

Taxon classificationPlantaeSapindalesRutaceae

(T.G.Hartley) Appelhans & W.L.Wagner comb. nov.

756EEBD4-C609-5F9A-8BB8-6581A1C18181

urn:lsid:ipni.org:names:77365254-1

Dutailliopsis gordonii T. G.Hartley, Adansonia III, 19: 210. 1997.

###### Type material.

**New Caledonia**: Riviére Bleue Reserve, 7 Oct. 1983, *McPherson 5844* (holotype CANB [CANB 345866.1]; isotypes MO [MO-251407!, MO-251408!], NOU [NOU006529!], P [P00094897!]).

##### ﻿4. Melicope lasioneura

Taxon classificationPlantaeSapindalesRutaceae

(Baill.) Baill. ex Guillaumin, Bull. Mus. Hist. Nat. (Paris) 26: 175. 1920.

89F9CC7B-26C4-54AC-8045-6FB561936970

Euodia lasioneura Baill., Adansonia 11: 179. 1874.
Melicope platystemon Baker f., J. Linn. Soc., Bot. 45: 280. 1921. Type: New Caledonia, Ignambi, Forest, 2500 ft., 12 Aug. 1914, *Compton 1717* (holotype BM [BM015145874!]).
Melicope leptophylla Guillaumin, Bull. Mus. Hist. Nat. (Paris), sér. 2, 10: 433. 1938. Type: New Caledonia, Mont Koghi, Apr. 1908, *LeRat & LeRat 2949* (holotype P [P00543950!]; isotype P [P00543951!]).

###### Type material.

**New Caledonia**: Forets au dessus d`Ouroué a l’embouchure du Dotio, Jul. 1871, *Balansa 3536* (holotype P [P00227569!]; isotypes NY [NY00055805!], P [P00543952!, P00543953!]).

##### ﻿5. Melicope pedicellata

Taxon classificationPlantaeSapindalesRutaceae

T. G.Hartley, Allertonia 8: 142. 2001.

D58C8FC9-6919-5194-8D9A-CEF2F64B3EF3

###### Type material.

**New Caledonia**: Pente nord du Plateau de Dogny, 11 Nov. 1966, *MacKee 15914* (holotype P [P00543949!]; isotype NOU [NOU006533!]).

##### ﻿6. Melicope vieillardii

Taxon classificationPlantaeSapindalesRutaceae

(Baill.) Baill. ex Guillaumin, Bull. Mus. Hist. Nat. (Paris) 26: 175. 1920.

B48FF1A3-D14E-5C5B-B244-533E598E3595

Euodia vieillardii Baill., Adansonia 11: 179. 1874, as “Evodia (Melicope)”.
Melicope montana Baker f., J. Linn. Soc., Bot. 45: 280. 1921. Type: New Caledonia, Tonine, mountain-top, 3500 ft., 30 Sept. 1914, *Compton 1937* (holotype BM [BM015145873!]).

###### Type material.

**New Caledonia**: Montagnes d. Balade, 1855–60, *Vieillard 296* (lectotype, designated by Hartley 2001, pg. 138: P [P00543945!]; isolectotype P [P00543944!]); Balade, bois de montagnes, 1855–60, *Vieillard 241* (syntype P [P00543943!]).

#### ﻿Melicopesect.Dutaillyea (Baill.) Appelhans & W.L.Wagner, stat. et comb. nov.

*Dutaillyea* Baill., Adansonia 10: 327. 1872.

*Comptonella* Baker f., J. Linn. Soc., Bot. 45: 281. 1921.

**Type species**: *Melicopetrifoliolata* (Baillon) Appelhans & W.L.Wagner, comb. nov.

**Note.** Ten species, two of which are subdivided into two varieties; endemic to New Caledonia.

##### ﻿1. Melicope amosensis

Taxon classificationPlantaeSapindalesRutaceae

(Guillaumin) Appelhans & W.L.Wagner comb. nov.

F0D5DC7A-8C06-5DD5-B116-5BDAAFF64627

urn:lsid:ipni.org:names:77365255-1

Sarcomelicope ? amosensis Guillaumin, Journ. Agric. Trop. Bot. Appl. 11: 94. 1964.
Dutaillyea amosensis (Guillaumin) T.G.Hartley, Adansonia 6: 33. 1984.

###### Type material.

**New Caledonia**: Col d´Amos versant de Ouégoa, 8 Jan. 1961, *MacKee 8005* (holotype P [P00057465!]; isotypes CANB [CANB120515!, CANB245628!], K [K000717645!], L [L0017829!]).

##### ﻿2. Melicope baudouinii

Taxon classificationPlantaeSapindalesRutaceae

(Baill.) Appelhans & W.L.Wagner comb. nov.

E3542F4E-E9F7-58E1-887D-20657718A7D4

urn:lsid:ipni.org:names:77365256-1

Euodia baudouinii Baillon, Adansonia 10: 326. 1871–1873, as “Evodia ?”.
Euodia hurlimannii Guillaumin, Mém. Mus. Natn. Hist. nat., sér. B, Bot. 8: 62. 1957, as “Evodia ?”. Type: New Caledonia, Fausse Yaté, 12 Jan. 1951, *Hürlimann 664* (holotype P [P00543993!]; isotype Z [Z-000025225!].
Comptonella baudouinii (Baill.) T.G.Hartley, Adansonia 4: 407. 1983.

###### Type material.

**New Caledonia**: 1865, *Baudouin s.n* . (holotype P [P00543994!]).

##### ﻿3. Melicope drupacea

Taxon classificationPlantaeSapindalesRutaceae

(Labill.) Appelhans & W.L.Wagner comb. nov.

DA8C0DA9-FFCE-5D9C-B275-C4FD746D1488

urn:lsid:ipni.org:names:77365257-1

Euodia drupacea Labill, Sertum Austro-Caled.: 73, tab. 74. 1825, as Evodia.
Euodia canalensis Baker f., J. Linn. Soc., Bot. 45: 282. 1921, as Evodia. Type: New Caledonia, Mt. Canala, 1914, *Compton 1196* (holotype BM [BM000798454!, BM000798455!], mounted on two sheets).
Comptonella drupacea (Labill.) Guillaumin, Bull. Soc. Bot. Fr. 85: 298, 299. 1938.

###### Type material.

**New Caledonia**: s.d., *Labillardiére s.n*. (holotype FI [FI0063746!]; isotypes G [G00087116!, G00087117!, G00087118!, G00087119!]).

##### 4.﻿ Melicope fruticosa

Taxon classificationPlantaeSapindalesRutaceae

(T.G.Hartley) Appelhans & W.L.Wagner comb. nov.

ACEDC9C7-EDD8-5843-BBC6-562355C7C72D

urn:lsid:ipni.org:names:77365258-1

Comptonella fruticosa T. G.Hartley, Adansonia 4: 406. 1983.

###### Type material.

**New Caledonia**: Voh, Crete sommitale du Mt. Katépahié, 600 m, 5 Apr. 1968, *MacKee 18630* (holotype P [P00543992!]).

##### 5.﻿ Melicope hartleyi

Taxon classificationPlantaeSapindalesRutaceae

Appelhans & W.L.Wagner nom. nov.

62EDC5D4-2FB7-5B64-B601-786C1343BFCC

urn:lsid:ipni.org:names:77365259-1

Dutaillyea sessilifoliola Guillaumin, Bull. Mus. Natn. Hist. nat., sér. 2, 4: 690. 1932.
Comptonella sessilifoliola (Guillaumin) T.G.Hartley, Adansonia 4: 411. 1983.

###### Type material.

**New Caledonia**: Val Suzon, 20 Jul. 1930, *Franc* s.n. (holotype P [P00543987!]).

**Note.** The specific epithet *sessilifoliola* is pre-empted in *Melicope*. The new species epithet honours Thomas G. Hartley, who revised the genera *Comptonella*, *Dutaillyea* and *Melicope* among many other Rutaceae genera.

##### ﻿6. Melicope homedeboense

Taxon classificationPlantaeSapindalesRutaceae

Appelhans & W.L.Wagner nom. nov.

A9CCF5FC-B385-595F-8478-65E5FECA8211

urn:lsid:ipni.org:names:77365260-1

Comptonella glabra T. G.Hartley, Adansonia 4: 399. 1983.

###### Type material.

**New Caledonia**: Taom, Mt. Homédeboa, 800–900 m, 16 Oct. 1969, *MacKee 20961* (holotype P [P00543991!]; isotype P [P00062001!]).

###### Note.

The specific epithet *glabra* is pre-empted in *Melicope*. *Melicopehomedeboense* is only known from its type locality on Mt. Homédeboa in maquis vegetation on serpentine soil.

##### ﻿7. Melicope lactea

Taxon classificationPlantaeSapindalesRutaceae

(Baker f.) Appelhans & W.L.Wagner comb. nov.

7D606432-8144-50A9-9149-A3B5849F30DC

urn:lsid:ipni.org:names:77365261-1

Euodia lactea Baker f., J. Linn. Soc., Bot. 45: 282. 1921, as Evodia. Comptonellalactea (Baker f.) T.G.Hartley, Adansonia 4: 408. 1983.

###### Type material.

**New Caledonia**: Nekando, 23 Oct. 1914, *Compton 2122* (holotype BM [BM000798431!]).

**7.1. Melicopelacteavar.lactea**

##### ﻿7.2. Melicope lactea var. poissonii

Taxon classificationPlantaeSapindalesRutaceae

(Guillaumin) Appelhans & W.L.Wagner comb. nov.

15E379D8-7F0F-5C68-94F5-C0E4D9FBF14D

urn:lsid:ipni.org:names:77365262-1

Dutaillyea poissonii Guillaumin, Bull. Soc. Bot. Fr. 85: 300. 1938.
Comptonella lactea (Baker f.) T.G.Hartley var. poissonii (Guillaumin) T.G.Hartley, Adansonia 4: 409. 1983.

###### Type material. New Caledonia:

s.d., *Pancher 275* (=*Vieillard 2454*) (lectotype, designated here: P [P00543990!]; isolectotypes MEL [MEL68395!], NY [NY00399955!], P [P00606571!, P00606572!, P00606573!]).

###### Note.

P00543990 is designated as the lectotype because it is the only specimen with precise locality information. The locality “Escarpement du Caugui” as written on the label is identical to the locality mentioned in the protologue (Mt. Koghi).

##### ﻿8. Melicope microcarpa

Taxon classificationPlantaeSapindalesRutaceae

(Perkins) Appelhans & W.L.Wagner comb. nov.

ADB34FC9-6321-566A-90D8-4EEA0941C42B

urn:lsid:ipni.org:names:77365263-1

Hedycarya microcarpa Perkins, in Engler, Pflanzenreich 4, 101, Nachträge (Heft 49): 4. 1911.
Comptonella albiflora Baker f., J. Linn. Soc., Bot. 45: 281, tab. 15, figs. 1–6. 1921. Type: New Caledonia, Ignambi, Forest, 3500 ft., 31. Jul. 1914, *Compton 1542* (holotype BM [BM015145875!]).
Comptonella microcarpa (Perkins) T.G.Hartley, Adansonia 4: 405. 1983.

###### Type material.

**New Caledonia**: s.d., *Caldwell s.n* . (holotype K [K000717646!]).

##### ﻿9. Melicope oreophila

Taxon classificationPlantaeSapindalesRutaceae

(Guillaumin) Appelhans & W.L.Wagner comb. nov.

1160EFC8-55E3-524A-B1D0-891EDCA09182

urn:lsid:ipni.org:names:77365264-1

Euodia oreophila Guillaumin, Bull. Soc. Bot. Fr. 85: 298. 1938, as “Evodia ?”.
Comptonella oreophila (Guillaumin) T.G.Hartley, Adansonia 4: 400. 1983.

###### Type material.

**New Caledonia**: Mont Mou, 14 Apr. 1870, *Balansa 2798a* (lectotype, designated by Hartley 1983, pg. 400: P [P00543988!,]; isolectotypes P [P00057462!, P00057463!]); Mont Arago, 27. Nov. 1869, *Balansa 1798* (syntypes A [A00105583!], P [P00227439! = Melicopeoreophilavar.longipes (Guillaumin) Appelhans & W.L.Wagner, P05214286! = Melicopeoreophilavar.longipes (Guillaumin) Appelhans & W.L.Wagner]); Mont Mou, Mar. 1870, *Balansa 2798* (syntypes A [A00105584! = Melicopeoreophilavar.longipes (Guillaumin) Appelhans & W.L.Wagner], P [P00227434!, P05214290!]).

**9.1. Melicopeoreophilavar.oreophila**

##### ﻿9.2. Melicope oreophila var. longipes

Taxon classificationPlantaeSapindalesRutaceae

(Guillaumin) Appelhans & W.L.Wagner comb. nov.

12A680B9-2511-5452-B611-098FBEAD8F85

urn:lsid:ipni.org:names:77365265-1

Dutaillyea longipes Guillaumin, Mém. Mus. Natn. Hist. nat., sér. B, Bot. 8: 63. 1957, as “Dutaillyea ?”.
Euodia fosteri Guillaumin, Bull. Mus. Natn. Hist. nat., sér 2, 29: 261. 1957, as “Evodia ?”. Types: New Caledonia, Edge of forest near summit, along trail to Plateau de Dogny, 20–21 May 1956, *Foster 79* (holotype P [P00057464!]; isotype UC [UC1078302!]).
Comptonella oreophila (Guillaumin) T.G.Hartley var. longipes (Guillaumin) T.G.Hartley, Adansonia 4: 402. 1983.

###### Type material.

**New Caledonia**: Mois de Mai, 25 Jul. 1951, *Baumann*-*Bodenheim 14252* (lectotype, designated by Hartley 1983, pg. 402: P [P00543989!,]; isolectotype Z [Z-000059487!]); Mois de Mai, 23 Jul. 1951, *Baumann*-*Bodenheim 14015* (syntypes P [P00227440!], Z [Z-000059488!]); Mois de Mai, 23 Jul. 1951, *Baumann*-*Bodenheim 14089* (syntypes G [G00074253!], P [P00227441!], Z [Z-000059492!, Z-000059493!]); Dzumac, 20 May 1951, *Hürlimann 429 [or 1429*] (syntypes L [L0017826!], P [P00606574!], Z [Z-000023326!, Z-000023327!]); Mt d. Sources, 7 Mar. 1951, *Hürlimann 982* (syntypes P [P00227442!], Z [Z-000059491!]).

###### Note.

*Dutaillyea ? longipes* Guillaumin var. ---, Mém. Mus. Natn. Hist. nat., sér. B, Bot. 8: 63. 1957 is not a published name as there is no epithet so it has no standing nomenclaturally and the cited specimen by Guillaumin for this variant is thus not a type.

##### ﻿10. Melicope trifoliolata

Taxon classificationPlantaeSapindalesRutaceae

(Baillon) Appelhans & W.L.Wagner comb. nov.

C8441C09-460A-50F2-97EF-2141DC673228

urn:lsid:ipni.org:names:77365266-1

Dutaillyea trifoliolata Baill., Adansonia 10: 328. 1872–1873.
Dutaillyea comptonii Baker f., J. Linn. Soc., Bot. 45: 283. 1921. Type: New Caledonia, Mont Panié, 29 Aug. 1914, *Compton 1819* (holotype BM [BM000798460!]).

###### Type material.

**New Caledonia**: Balade, 1855–1860, *Vieillard 1033* (lectotype, designated by Hartley 1984, pg. 30: P [P00543984!]; isolectotypes P [P00543983!, P00543985!]).

#### ﻿Melicopesect.Lepta (Lour.) T.G.Hartley, Allertonia 8: 71. 2001.

*Lepta Lour.* Fl. Cochinch.: 82. 1790.

*Boninia Planch*. Ann. Sci. Nat., Bot., sér. 5, 14: 309. 1871.

*Tractocopevodia Raizada & V. Naray.*, Indian Forester 72: 275. 1946.

*Evodiella B. L. Linden*, Nova Guinea, n.s., 10: 145. 1959).

**Type species**: *Melicopepteleifolia* (Champ. ex Benth.) T.G.Hartley.

**Notes.** 102 species; distributed from India to the Japanese Bonin Islands, throughout Malesia and the South Pacific until Tonga; also on Madagascar and the Mascarene Islands.

No new species have been published in this section since Hartley’s monograph in 2001, and the only taxonomic change was the correction of the name *M.confusa* (Merr.) P.S.Liu to *M.frutescens* (Blanco) Appelhans & J.Wen (Appelhans and Wen 2016). In order not to repeat the work done by Hartley (2001), we only list the names of species and refer to Hartley (2001) for lists of synonyms, typification and distribution ranges.

*Melicopeaccedens* (Blume) T.G.Hartley, *Melicopeacuminata* (Merr.) T.G.Hartley, *Melicopeaffinis* T.G.Hartley, *Melicopealpestris* T.G.Hartley, *Melicopeanomala* (Lauterb.) T.G.Hartley, *Melicopebakeri* T.G.Hartley, *Melicopebalankazo* (H.Perrier) T.G.Hartley, *Melicopebelahe* (Baill.) T.G.Hartley, *Melicopebenguetensis* (Elmer) T.G.Hartley, *Melicopeblancoi* T.G.Hartley, *Melicopebonwickii* (F.Muell.) T.G.Hartley, *Melicopeborbonica* (Bory) T.G.Hartley, *Melicopebuennemeijeri* T.G.Hartley, *Melicopeburmahia* (Raizada & K.Naray.) T.G.Hartley, *Melicopebuwaldae* T.G.Hartley, *Melicopecalycina* T.G.Hartley, *Melicopecelebica* T.G.Hartley, *Melicopechapelieri* (Baill.) T.G.Hartley, *Melicopechunii* (Merr.) T.G.Hartley, *Melicopeclemensiae* T.G.Hartley, *Melicopecoodeana* T.G.Hartley, *Melicopecorneri* T.G.Hartley, *Melicopecrassifolia* (Merr.) T.G.Hartley, *Melicopecrassiramis* (K.Schum.) T.G.Hartley, *Melicopecravenii* T.G.Hartley, *Melicopecrispula* (Merr. & L.M.Perry) T.G.Hartley, *Melicopecucullata* (Gillespie) A.C.Sm., *Melicopedecaryana* (H.Perrier) T.G.Hartley, *Melicopedenhamii* (Seem.) T.G.Hartley, *Melicopediscolor* (Baker) T.G.Hartley, *Melicopedoormani-montis* (Lauterb.) T.G.Hartley, *Melicopedubia* (Merr.) T.G.Hartley, *Melicopeelleryana* (F.Muell.) T.G.Hartley, *Melicopeeriophylla* (Merr. & L.M.Perry) T.G.Hartley, *Melicopeeuneura* (Miq.) T.G.Hartley, *Melicopeexuta* T.G.Hartley, *Melicopefatraina* (H.Perrier) T.G.Hartley, *Melicopefellii* T.G.Hartley, *Melicopefloribunda* (Baker) T.G.Hartley, *Melicopeforbesii* (Baker f.) T.G.Hartley, *Melicopefrutescens* (Blanco) Appelhans & J.Wen, *Melicopeglabella* T.G.Hartley, *Melicopeglabra* (Blume) T.G.Hartley, *Melicopeglomerata* (Craib) T.G.Hartley, *Melicopegrisea* (Planch.) T.G.Hartley, *Melicopehayesii* T.G.Hartley, *Melicopehiepkoi* T.G.Hartley, *Melicopehookeri* T.G.Hartley, *Melicopeidiocarpa* T.G.Hartley, *Melicopeimprovisa* T.G.Hartley, *Melicopeincana* T.G.Hartley, *Melicopeirifica* (Coode) T.G.Hartley

*Melicopejonesii* T.G.Hartley, *Melicopekainantuensis* T.G.Hartley, *Melicopekjellbergii* T.G.Hartley, *Melicopekostermansii* T.G.Hartley, *Melicopelatifolia* (DC.) T.G.Hartley, *Melicopelaxa* (Elmer) T.G.Hartley, *Melicopelunu-ankenda* (Gaertn.) T.G.Hartley, *Melicopemacrocarpa* (King) T.G.Hartley, *Melicopemadagascariensis* (Baker) T.G.Hartley, *Melicopemagnifolia* (Baill.) T.G.Hartley, *Melicopemaliliensis* T.G.Hartley, *Melicopemaxii* T.G.Hartley, *Melicopemegastigma* T.G.Hartley, *Melicopemicrococca* (F.Muell.) T.G.Hartley, *Melicopemindorensis* T.G.Hartley, *Melicopemoluccana* T.G.Hartley, *Melicopemonticola* T.G.Hartley, *Melicopeneglecta* T.G.Hartley, *Melicopenishimurae* (Koidz.) T.Yamaz., *Melicopeobscura* (Cordem.) T.G.Hartley, *Melicopeobtusifolia* (DC.) T.G.Hartley, *Melicopepachyphylla* (King) T.G.Hartley, *Melicopepachypoda* (Lauterb.) T.G.Hartley, *Melicopepahangensis* T.G.Hartley, *Melicopepalawensis* (Lauterb.) T.G.Hartley, *Melicopepauciflora* T.G.Hartley, *Melicopependula* T.G.Hartley, *Melicopepeninsularis* T.G.Hartley, *Melicopepergamentacea* (Elmer) T.G.Hartley, *Melicopepteleifolia* (Champ. ex Benth.) T.G.Hartley, *Melicopepulgarensis* (Elmer) T.G.Hartley, *Melicopequadrilocularis* (Hook. & Arn.) T.G.Hartley, *Melicoperamuliflora* T.G.Hartley, *Melicoperhytidocarpa* (Merr. & L.M.Perry) T.G.Hartley, *Melicoperigoensis* T.G.Hartley, *Melicoperubra* (Lauterb. & K.Schum.) T.G.Hartley, *Melicopesambiranensis* (H.Perrier) T.G.Hartley, *Melicopeschraderi* (Lauterb.) T.G.Hartley, *Melicopesegregis* (Cordem.) T.G.Hartley, *Melicopesemecarpifolia* (Merr.) T.G.Hartley, *Melicopesessilifoliola* (Merr.) T.G.Hartley, *Melicopesteenisii* T.G.Hartley, *Melicopesubunifoliolata* (Stapf) T.G.Hartley, *Melicopetimorensis* T.G.Hartley, *Melicopetrichantha* (Lauterb.) T.G.Hartley, *Melicopetrichopetala* (Lauterb.) T.G.Hartley, *Melicopetsaratananensis* (Capuron) T.G.Hartley, *Melicopevillosa* (Merr.) T.G.Hartley, *Melicopeviticina* (Wall. ex Kurz) T.G.Hartley, *Melicopezambalensis* (Elmer) T.G.Hartley

#### ﻿Melicopesect.Melicope

*Astorganthus* Endl. ex Hook., Cat. Hort. Vindob. 2: 196. 1843.

*Entoganum* Banks ex Gaertn., Fruct. Sem. Pl. i. 331. t. 68. 1788.

**Type species**: *Melicopeternata* J.R.Forst. & G.Forst.

**Note.** Five or six species, as delimited by Appelhans et al. 2014c; distributed in New Zealand, Kermadec Islands, Society Islands, Austral Islands.

##### ﻿1. Melicope balgooyi

Taxon classificationPlantaeSapindalesRutaceae

Appelhans, W.L.Wagner & K.R.Wood, PhytoKeys 39: 78. 2014.

44D2F1F0-5728-58DB-A902-81D175EAA9DA

###### Type material.

**Austral Islands**: Rapa Iti, Maii, below rim near Pokumaru, 29 Apr. 2002, *K.R.Wood 9727* (holotype PTBG [PTBG-041326!]; isotype NY!).

##### ﻿2. Melicope lucida

Taxon classificationPlantaeSapindalesRutaceae

(A.Gray) A.C.Sm., J. Arnold Arbor. 32: 249. 1951.

F5452A65-574B-5074-A084-D9BBEDC2D16F

Pelea lucida A. Gray, Proc. Amer. Acad. Arts 3: 51. 1853., Bot. U.S. Expl. Exped. 1: 348. 1854., Atlas, tab. 34, Β. 1857, as “Pelea ?”.
Melicope tahitensis var. glabrata Nadeaud, Énum. Pl. Tahiti 76. 1873. Type: Society Islands, Tahiti, Pinai et Rereaoe, s.d., *J.Nadeaud 472 p.p*. (holotype P [P00646095!]).
Melicope auriculata Nadeaud, Énum. Pl. Tahiti 76. 1873. Types: Society Islands, Tahiti, s.d., *J.Nadeaud 473 p.p*. (lectotype, designated by Hartley 2001, pg. 106, P [P00646061!]); Society Islands, Tahiti, en Teumupuaa [Teumopua] in Taiarapu [Teahupoo?], s.d., *J.Nadeaud 473 p.p*., as var. B (syntypes P [P00646075!], G [G00096087!]); Society Islands, Tahiti, Orofero vallée, s.d., *J.Nadeaud 473 p.p*., as var. A (syntypes P [P00646076!, P00646077!]); Society Islands, Tahiti, monte Mahutaa, s.d., *J.Nadeaud 473 p.p*., as var. C (not located).
Melicope leguminosa Nadeaud, Énum. Pl. Tahiti 76. 1873. Types: Society Islands, Tahiti, montagnes de Mahaena à Tuumatairiri, 12 Jul. 1857, *J.Nadeaud 474* (holotype, P [P00646079!]). Note: Two additional Nadeaud specimens have been located for this taxon (G00096088!, P00646060!). We do not consider the specimen at G as original material, because it was collected on July 15^th^, 1857, whereas the date July 12^th^, 1857 is mentioned in the protologue. The specimen at P might represent additional authentic material, but does not contain precise label information about the locality (Ile de Tahiti) and date. Hartley annotated this specimen as the holotype, but we consider P00646079 as the holotype because it is the only specimen with the matching date.
Euodia auriculata (Nadeaud) Drake, Fl. Ins. Pacif. 19. tab. 4. 1886. Type: Based on Melicopeauriculata Nadeaud.
Euodia lucida (A.Gray) Drake, Ill. Fl. Ins. Pacif.: 134. 1890, as “Euodia ?”.
Euodia leguminosa (Nadeaud) Drake, Ill. Fl. Ins. Pacif. 132. 1890. Type: Based on Melicopeleguminosa Nadeaud.

###### Type material.

“**Samoa**: Mts. of Tutuila” [actually Society Islands], 1838–1842, *U.S. Expl. Exped. s.n*., (holotype US [US00101483!]; isotypes GH [GH00105702!], NY [NY00055937!]).

###### Note.

Hartley (2001) argues that the type location is probably erroneous since the species is not known from Samoa and that it presumably originates from Tahiti.

##### ﻿3. Melicope mantellii

Taxon classificationPlantaeSapindalesRutaceae

Buchanan, Trans. & Proc. New Zealand Inst. 3: 212. 1871.

7BCC0D20-EB28-5299-9F33-521898F92B45

Melicope × tersimplex Allan, Genetica 9: 145. 1927.
Melicope ternata var. mantellii (Buchanan) Kirk, Forest Fl. New Zealand: 119, tab. 67. 1889.

###### Type material.

**New Zealand**: s.d., *Buchanan s.n*. (lectotype, designated here, WELT [WELT.SP029403!]); s.d., *Buchanan s.n*. (syntype WELT [WELT.SP029749!]). Note: The two specimens bear no precise label information and no dates, but both have been collected by Buchanan. We selected SP029403 as the lectotype because it is from Buchanan’s herbarium, while the other specimen belongs to the T. Kirk herbarium.

###### Note.

*Melicopemantellii* is likely a hybrid of *M.simplex* and *M.ternata* (Cheeseman 1906; Cockayne and Allan 1934; Fujita 1961; see also Hartley 2001). We follow Hartley (2001) and treat it tentatively as a separate species, because genetic or phylogenomic analyses that support the hybrid origin of *M.mantellii* are lacking. It is listed as a hybrid in the *Flora of New Zealand* (Allan 1961).

##### ﻿4. Melicope simplex

Taxon classificationPlantaeSapindalesRutaceae

A. Cunn., Ann. Nat. Hist. 3: 315. 1839.

E6085914-34EA-5EA3-B58D-31B3261BB85A

Melicope parvula Buchanan, Trans. & Proc. New Zealand Inst. 20: 255. 1888. Type: New Zealand, s.d., Buchanan s.n., (holotype WELT!, in Buchanan’s bound herbarium).

###### Type material.

**New Zealand**: 1826, *Cunningham 57* (holotype K [K000340051!]; isotypes BR [BR0000005640843!], W [W0325222!]). Note: Three additional Cunningham specimens have been located (BR0000005640515!, K000340052!, WELT.SP079517!), which might represent original material of *Melicopesimplex*. However, they do not contain a date.

##### ﻿5. Melicope tahitensis

Taxon classificationPlantaeSapindalesRutaceae

Nadeaud, Énum. Pl. Tahiti 75. 1873.

301939B9-D1E3-5209-90DD-0CAFF9CABAB6

Melicope tahitensis var. puberula Nadeaud, Énum. PI. Tahiti 76. 1873, nom. invalid.
Euodia sericea Drake, Ill. Fl. Ins. Pacif.: 15, tab. 2. 1886. Type: Society Islands, Tahiti, 1847, Vesco s.n. (holotype P [P00978590!, P00978591!, holotype mounted on two sheets as indicated by the remarks “1/2” and “2/2” on the sheets]). Note: The species name Euodiasericea is not mentioned on the label, but the handwritten description and the morphology match with the description in the protologue. There is a remark “dupl. PAP” on the label, but a specimen could not be located at PAP (pers. comm. Tamara Maric; 16 March 2024).
Euodia nodulosa Drake, Ill. Fl. Ins. Pacif.: 17, tab. 3. 1886. Type: Society Islands, Tahiti, 1847, Vesco s.n. (holotype P [P00978592!]). Note: The species name Euodianodulosa is not mentioned on the label, but the handwritten description and the morphology match with the description in the protologue.
Euodia nadeaudii Drake, Ill. Fl. Ins. Pacif.: 132. 1890.
Melicope diversifolia Guillaumin, Bull. Mus. Hist. Nat. (Paris), sér. 2, 14: 287. 1942. Type: New Caledonia, Balade, 1855–60, *Vieillard 295*, (holotype P [P00543962!]; isotypes P [P00227568!, P00543959!, P00543960!, P00543961!]). Note: Hartley (2001) notes that the type was probably collected in Tahiti instead of New Caledonia. The species does not occur on New Caledonia.

###### Type material.

**Society Islands**: Tahiti, s.d., *J. Nadeaud 472 p.p*. (holotype P [P00639236!]; isotype P [P00646057!]).

##### ﻿6. Melicope ternata

Taxon classificationPlantaeSapindalesRutaceae

J.R.Forst. & G.Forst., Char. Gen. Pl. 28, tab. 28. 1775; ed. 2, 56, tab. 28. 1776.

457FACC2-4F3F-5FA5-8777-5F1C301AC3A3

Entoganum laevigatum Banks ex Gaertn., Fruct. Sem. Pl. i: 331, tab. 68, fig. 6. 1788. Type: New Zealand, prope Tolago, 1768–1770, Banks & Solander s.n. (holotype BM [BM015145871!]; isotype US [US00610724!]).
Melicope ternata var. grandis Cheeseman, Trans. & Proc. New Zealand Inst. 20: 166. 1888. Type: Kermadec Group, Sunday Island, Aug. 1887, Cheeseman s.n. (holotype AK [AK229826!, AK229827!, AK5029!, holotype mounted on three sheets]; isotype K [K000717402!]).

###### Type material.

**New Zealand**: s.d., *J.R. & G.Forster s.n*. (lectotype, designated by Hartley 2001, pg. 91: K [K000717403!]); additional potential original material (Nicolson and Fosberg 2003): New Zealand, s.d., *W. Anderson s.n*. (BM); New Zealand, s.d., *Forster? s.n*. (LINN [LINN-HS676-1!]); New Zealand, s.d., *Forster s.n*. (S); New Zealand, s.d., *Forster? s.n*. (S); Insulis Maris Pacifici, s.d., *A. Sparrman s.n*. (UPS [UPS-T 9207 (V-009207)]. Note: Hartley annotated the Kew specimen as an isotype, but he noted (Hartley 2001) that he had only seen a single specimen, so that this has to be the lectotype specimen.

###### Note.

*Melicopeternata* J.R.Forst. & G.Forst. is the type species of the genus.

#### ﻿Melicopesect.Pelea (A.Gray) Hook.f., in Bentham & Hooker, Gen. PI. 1: 296. 1862.

*Pelea* A.Gray, Proc. Amer. Acad. Arts 3: 50. 1853.

**Type species.***Melicopeclusiifolia* (A.Gray) T.G.Hartley & B.C.Stone

**Notes.** 87 species; distributed from New Guinea throughout most Pacific Archipelagos until the Hawaiian Islands and the Marquesas Islands; one widespread species [*M.triphylla*] also found further westward until Borneo, the Philippines, Taiwan and the Japanese Ryukyu Islands; the center of distribution is the Hawaiian Islands.

Since Hartley’s monograph in 2001, three new species have been published (Wood et al. 2016, 2017, 2024), the four species of *Platydesma* have been merged into the section (Appelhans et al. 2017), and five species from New Caledonia have been removed from the section (this study). We refer to Hartley (2001) for lists of synonyms, typification and distribution ranges.

*Melicopeaberrans* T.G.Hartley, *Melicopeadscendens* (H.St.John & E.P.Hume) T.G.Hartley & B.C.Stone, *Melicopealba* Lauterb., *Melicopealbiflora* (Rech.) T.G.Hartley, *Melicopeaneura* (Lauterb.) T.G.Hartley, *Melicopeanisata* (H.Mann) T.G.Hartley & B.C.Stone, *Melicopeballoui* (Rock) T.G.Hartley & B.C.Stone, *Melicopebarbigera* A.Gray, *Melicopeboweriana* (Christoph.) T. G. Hartley,

*Melicopebrassii* T.G.Hartley, *Melicopechristophersenii* (H.St.John) T.G.Hartley & B.C.Stone, *Melicopecinerea* A.Gray, *Melicopeclusiifolia* (A.Gray) T.G.Hartley & B.C.Stone, *Melicopeconjugata* T.G.Hartley, *Melicopecornuta* (Hillebr.) Appelhans, K.R.Wood & W.L.Wagner, *Melicopecruciata* (A.Heller) T.G.Hartley & B.C.Stone, *Melicopedegeneri* (B.C.Stone) T.G.Hartley & B.C.Stone, *Melicopedurifolia* (K.Schum.) T.G.Hartley, *Melicopeelliptica* A.Gray, *Melicopefatuhivensis* (F.Br.) T.G.Hartley & B.C.Stone, *Melicopefeddei* (H.Lév.) T.G.Hartley & B.C.Stone, *Melicopehaleakalae* (B.C.Stone) T.G.Hartley & B.C.Stone, *Melicopehaupuensis* (H.St.John) T.G.Hartley & B.C.Stone, *Melicopehawaiensis* (Wawra) T.G.Hartley & B.C.Stone, *Melicopehiiakae* (B.C.Stone) T.G.Hartley & B.C.Stone, *Melicopehivaoaensis* J.Florence, *Melicopehosakae* (H.St.John) W.L.Wagner & R.K.Shannon, *Melicopeinopinata* J.Florence, *Melicopeiolensis* K.R.Wood, Lorence & W.L.Wagner, *Melicopekaalaensis* (H.St.John) T.G.Hartley & B.C.Stone, *Melicopekavaiensis* (H.Mann) T.G.Hartley & B.C.Stone, *Melicopeknudsenii* (Hillebr.) T.G.Hartley & B.C.Stone, *Melicopelamii* Lauterb., *Melicopelauterbachii* T.G.Hartley, *Melicopelobocarpa* (F.Muell.) T.G.Hartley, *Melicopelydgatei* (Hillebr.) T.G.Hartley & B.C.Stone, *Melicopemacrophylla* Merr. & L.M.Perry, *Melicopemacropus* (Hillebr.) T.G.Hartley & B.C.Stone, *Melicopemakahae* (B.C.Stone) T.G.Hartley & B.C.Stone, *Melicopemolokaiensis* (Hillebr.) T.G.Hartley & B.C.Stone, *Melicopemucronulata* (H.St.John) T.G.Hartley & B.C.Stone, *Melicopemunroi* (H.St.John) T.G.Hartley & B.C.Stone, *Melicopenealae* (B.C.Stone) T.G.Hartley & B.C.Stone, *Melicopenubicola* T.G.Hartley, *Melicopenukuhivensis* (F.Br.) T.G.Hartley & B.C.Stone, *Melicopeoahuensis* (H.Lév.) T.G.Hartley & B.C.Stone, *Melicopeobovata* (H.St.John) T.G.Hartley & B.C.Stone, *Melicopeoppenheimeri* K.R.Wood, Appelhans & W.L.Wagner, *Melicopeorbicularis* (Hillebr.) T.G.Hartley & B.C.Stone, *Melicopeovalis* (H.St.John) T.G.Hartley & B.C.Stone, *Melicopeovata* (H.St.John & E.P.Hume) T.G.Hartley & B.C.Stone, *Melicopepallida* (Hillebr.) T.G.Hartley & B.C.Stone, *Melicopepaniculata* (H.St.John) T.G.Hartley & B.C.Stone, *Melicopepeduncularis* (H.Lév.) T.G.Hartley & B.C.Stone, *Melicopeperlmanii* J.Florence, *Melicopepolyadenia* Merr. & L.M.Perry, *Melicopeponapensis* Lauterb., *Melicopepseudoanisata* (Rock) T.G.Hartley & B.C.Stone, *Melicopepuberula* (H.St.John) T.G.Hartley & B.C.Stone, *Melicopequadrangularis* (H.St.John & E.P.Hume) T.G.Hartley & B.C.Stone, *Melicoperadiata* (H.St.John) T.G.Hartley & B.C.Stone, *Melicopereflexa* (H.St.John) T.G.Hartley & B.C.Stone, *Melicoperemyi* (Sherff) Appelhans, K.R.Wood & W.L.Wagner, *Melicoperetusa* (A.Gray) T.G.Hartley, *Melicoperevoluta* J.Florence, *Melicoperichii* (A.Gray) T.G.Hartley, *Melicoperobbinsii* T.G.Hartley, *Melicoperostrata* (Hillebr.) Appelhans, K.R.Wood & W.L.Wagner, *Melicoperotundifolia* (A.Gray) T.G.Hartley & B.C.Stone, *Melicopesaint-johnii* (E.P.Hume) T.G.Hartley & B.C.Stone, *Melicopesandwicensis* (Gaudich. ex Hook. & Arn.) T.G.Hartley & B.C.Stone, *Melicopesavaiensis* T.G.Hartley, *Melicopesessilis* (H.Lév.) T.G.Hartley & B.C.Stone, *Melicopespathulata* A.Gray, *Melicopesterrophylla* Merr. & L.M.Perry, *Melicopestonei* K.R.Wood, Appelhans & W.L.Wagner, *Melicopesulcata* T.G.Hartley, *Melicopetekaoensis* T.G.Hartley, *Melicopetriphylla* (Lam.) Merr., *Melicopevatiana* (Setch.) T.G.Hartley, *Melicopeversteeghii* T.G.Hartley, *Melicopevinkii* T.G.Hartley, *Melicopevolcanica* (A.Gray) T.G.Hartley & B.C.Stone, *Melicopewaialealae* (Wawra) T.G.Hartley & B.C.Stone, *Melicopewailauensis* (H.St.John) T.G.Hartley & B.C.Stone, *Melicopewawraeana* (Rock) T.G.Hartley & B.C.Stone, *Melicopezahlbruckneri* (Rock) T.G.Hartley & B.C.Stone

#### ﻿Melicopesect.Picrella (Baill.) Appelhans & W.L.Wagner, stat. et comb. nov.

*Picrella* Baill., Adansonia 10: 150. 1871.

*Zieridium* Baill., Adansonia 10: 303. 1872.

Melicopesect.Vitiflorae T.G.Hartley *pro maj*. *Parte*, Allertonia 8: 68. 2001.

**Type species**: *Melicopetrifoliata* (Baill.) Appelhans & W.L.Wagner, comb. nov.

**Notes.** Ten species, one of which is subdivided into three varieties; distributed in the South Pacific [Lord Howe Island, Norfolk Island, New Caledonia, Vanuatu, Fiji, Cook Islands, Society Islands, Austral islands].

All species of Melicopesect.Vitiflorae except its type species *M.vitiflora* are part of this section. *Melicopevitiflora* has been shown to be most closely related to the monotypic *Pitaviaster* T.G.Hartley and *Euodiapubifolia* T.G.Hartley (Appelhans et al. 2014b)

##### ﻿1. Melicope bracteata

Taxon classificationPlantaeSapindalesRutaceae

(Nadeaud) S.L. Welsh, Fl. Societensis 255. 1998.

A1D38043-6267-5A4A-B7CC-7A1BAD4C4AD2

Euodia bracteata Nadeaud, Énum. Pl. Tahiti 76. 1873.
Euodia tahitensis Nadeaud, Énum. Pl. Tahiti 77. 1873, as Euodiatahitensisvar.peninsulae Nadeaud, Énum. Pl. Tahiti 77. 1873, nom. invalid. Type: Society Islands, Tahiti, Mataoae, in Teumupuaa, Taiarapu, 6 Jul. 1858, *Nadeaud 476 p.p*. (holotype P [P00639246!]; isotypes G [G00096022!], P [P00639244!, P00641431!]).
Euodia tahitensis var. brachiata Nadeaud, Énum. Pl. Tahiti 77. 1873. Type: Society Islands, Tahiti, Mont. Mahutaa, en vallée de Orofero, s.d., Nadeaud s.n. / 476 p.p. (holotype P [P00646093!]; isotype P [P00646064!]).
Euodia tahitensis var. ovata Nadeaud, Énum. Pl. Tahiti 77. 1873. Type: Society Islands, Tahiti, in Orofero valle ad Punaaruu, s.d., Nadeaud s.n. / 476A (holotype P [P00646094!]; isotype P [P00639235!]). Note: Hartley annotated the isotype specimen as the holotype, but it does not contain the original label.
Euodia emarginata Drake, Ill. Fl. Ins. Pacif. 20, tab. V. 1886. Type: Society Islands, Tahiti, montagn. Taravao, 1847, *Lépine 211* (holotype: P [P00639242!]; isotype G [G00096021!]).
Euodia lepinei Baill. ex Drake, Ill. Fl. Ins. Pacif. 22, tab. VI. 1886. Type: Society Islands, Tahiti, gorges de Papénoo, 1847, *Lépine 80* (holotype P [P00639238!]).
Euodia brachiata (Nadeaud) Drake, Ill. Fl. Ins. Pacif. 131. 1890. Type: Based on Euodiatahitensisvar.brachiata Nadeaud.
Melicope brachiata (Nadeaud) S. L. Welsh, Fl. Societensis 254. 1998. Type: Based on Euodiatahitensisvar.brachiata Nadeaud.
Melicope emarginata (Drake) S.L.Welsh, Fl. Societensis 255. 1998. Type: Based on Euodiaemarginata Drake.
Melicope tiarapuensis S.L.Welsh, Fl. Societensis 255. 1998. Type: Based on Euodiatahitensis Nadeaud.

###### Type material.

**Society Islands**: Tahiti, crêtes de Pirae à l´Aorai, s.d., *Nadeaud 475* (holotype P [P00646065!]; isotypes G [G00096019!, G00096020!], P [P00639243!]). Note: Hartley annotated the specimen P00639243 as the holotype, but it does not contain the original label and is from the herbarium of Emmanuel Drake del Castillo. Specimen P00646065 is from Jean Nadeaud’s herbarium, contains the original label with the precise locality and the word “ipse” (=himself) is mentioned as the author name as in the protologue. We are therefore convinced that this specimen is the holotype and Hartley might not have seen this specimen.

##### ﻿2. Melicope capillacea

Taxon classificationPlantaeSapindalesRutaceae

(Gillespie) A.C. Sm., Fl. Vit. Nov. 3: 506. 1985.

45DB4BB1-8096-51E8-A447-102074C1E538

Euodia capillacea Gillespie, Bernice P. Bishop Mus. Bull. 91: 10, fig. 10. 1932.

###### Type material.

**Fiji**: Viti Levu, vicinity of Nandarivatu, 2 miles along Mba road, 25 Nov. 1927, *Gillespie 4046* (holotype BISH [BISH1004814!]; isotypes GH [GH00105670!], UC [UC448530!]).

##### ﻿3. Melicope erromangensis

Taxon classificationPlantaeSapindalesRutaceae

T. G.Hartley, Allertonia 8: 133. 2001.

14580781-86D0-5F15-BB62-05BFDCFBB375

###### Type material.

**Vanuatu**: Erromango, Potnarhoin, pente N du Santop, 19 Jul. 1984, *Sam 198* (holotype CANB [CANB497818!]; isotypes NOU [NOU082033!, NOU082034!]).

##### ﻿4. Melicope glandulosa

Taxon classificationPlantaeSapindalesRutaceae

(T.G.Hartley) Appelhans & W.L.Wagner comb. nov.

D4065CF6-75BE-55F6-98D4-4D0D10BA14A1

urn:lsid:ipni.org:names:77365267-1

Picrella glandulosa T. G.Hartley, Adansonia III 25: 253. 2003.

###### Type material.

**New Caledonia**: Yaté road, valley after Col des Dalmates, 26 Jun. 1955, *MacKee 2660* (holotype P [P00543931!]; isotypes L [L.2119086!], UC [UC1300557!], US [US00731458!]).

##### ﻿5. Melicope ignambiensis

Taxon classificationPlantaeSapindalesRutaceae

(Guillaumin) Appelhans & W.L.Wagner comb. nov.

86D0D75E-B36E-53FB-B14A-4858ACF2D2B1

urn:lsid:ipni.org:names:77365268-1

Euodia ignambiensis Guillaumin, in Sarasin & Roux, Nova Caledonia, Bot. 1: 161. 1920.
Picrella ignambiensis (Guillaumin) T.G.Hartley & Mabb., Adansonia III 25: 256. 2003.

###### Type material.

**New Caledonia**: Mt. Ignambi, 4 Oct. 1911, *Sarasin 186* (holotype P [P00222156!]; isotypes BAS [BAS-00001210!], Z [Z-000023336!]).

##### ﻿6. Melicope laevis

Taxon classificationPlantaeSapindalesRutaceae

T. G.Hartley, Allertonia 8: 132. 2001.

F5971BBC-391A-55D1-AD18-CE010F4A1862

###### Type material.

**Vanuatu**: Espiritu Santo, Cap Cumberland, crête en direction du Voutmélé, 3 Aug. 1979, *Veillon 4013* (holotype CANB [CANB285904!]; isotypes NOU [NOU082031!, NOU082032!]).

##### ﻿7. Melicope littoralis

Taxon classificationPlantaeSapindalesRutaceae

(Endl.) T.G.Hartley, Kew Bull. 45: 250. 1990.

D2CDE2A0-BCC8-5605-9866-6F4A17B501C8

Euodia littoralis Endl., Prodr. Fl. Norfolk: 86. 1833.
Ampacus littoralis (Endl.) Kuntze, Revis. Gen. Pl. 1: 98. 1891, as A.litoralis.

###### Type material.

**Australia**: Norfolk Island, Ansons Bay, s.d., *Bauer 157* (holotype W [W0046197!]). Note: W0046198 is probably a part of the holotype mounted on a second sheet.

##### ﻿8. Melicope margaretae

Taxon classificationPlantaeSapindalesRutaceae

(F.Br.) T.G.Hartley, Allertonia 8: 136. 2001.

F3A5F6B7-F586-5881-B5E7-F6B2E4D6670A

Euodia margaretae F.Br., Bernice P. Bishop Mus. Bull. 130: 130, fig. 20, m, n. 1935.

###### Type material.

**Austral Islands**: Rapa Nui, Maungaaeae, 19 Oct. 1921, *A. M. Stokes 352* (holotype BISH [BISH1004877!]). Note: The specimen has been annotated as the isotype (by T. G. Hartley on March 10, 1989, by K. Kami in June 1997) and Hartley (2001) mentioned another specimen at BISH as the holotype, which he had not seen. Another specimen could not be located at BISH (pers. comm. Timothy Gallaher, 18 March 2024), so that BISH1004877 has to be the holotype. The confusion about a second specimen could be because of the remark “1^st^ sheet; 2^nd^ sheet is no. 370” on the specimen. This second sheet (BISH1273707!) has been collected by J. F. G. Stokes during the same journey on 26 October 1921 and is not mentioned in the protologue.

##### ﻿9. Melicope polybotrya

Taxon classificationPlantaeSapindalesRutaceae

(C.Moore & F.Muell.) T.G.Hartley, Kew Bull. 45: 250. 1990.

D7223860-2A9A-59BD-815A-23B9CBE904F9

Euodia polybotrya C. Moore & F. Muell., Fragm. 7: 143. 1871.
Ampacus polybotrys (C. Moore & F. Muell.) Kuntze, Revis. Gen. Pl. 1: 98. 1891.

###### Type material.

**Australia**: Lord Howe Island, Mt. Lidgebird, s.d. *Moore & Carron 41* (lectotype, designated by Hartley 2001, pg. 136: MEL [MEL502450!]); Lord Howe Island, s.d., *Moore 42* (syntypes MEL [MEL502451!], K [K000717407!]). Note: There is another *Moore 42* specimen at K (K000717408!), but it bears the date “2/72”, which is later than the publication of the species and the specimen is therefore not suited as original material.

##### ﻿10. Melicope trifoliata

Taxon classificationPlantaeSapindalesRutaceae

(Baill.) Appelhans & W.L.Wagner comb. nov.

6A586365-E6AF-52FE-A548-3086F8E2C7ED

urn:lsid:ipni.org:names:77365269-1

Picrella trifoliata Baill., Adansonia 10: 150, plate X. 1871.
Helietta trifoliata (Baill.) Mabb., Plant-book, corr. repr.: 707. 1989.
Euodia pseudo-obtusifolia Guillaumin, Bull. Mus. Natl. Hist. Nat. 26: 176. 1920. Type: New Caledonia, s.d., *Le Rat & Le Rat 732* (holotype P [P00543998!]).
Zieridium pseudo-obtusifolium (Guillaumin) Guillaumin, Bull. Soc. Bot. France 85: 299. 1938. Type: Based on Euodiapseudo-obtusifolia Guillaumin
Zieridium melicopifolium Guillaumin, Bull. Soc. Bot. France 85: 299. 1938. Type: New Caledonia, 1868–1870, *Balansa 1799* (holotype P [P00543903!, P00543904!, mounted on two sheets]). Note: Hartley and Mabberley (2003) mentioned an additional isotype at K, but it could not be located (pers. comm. Alison Moore, 6 March 2024).

###### Type material.

**France**: cult. Jardin des Plantes, Paris, Aug. 1871, *Anon*. *s.n*. (holotype P-Baill.!); cult. Jardin des Plantes, Paris, Oct. 1871, *Anon*. *s.n*. (topotype P [P00259629!]). Note: The origin of this cultivated plant from the Botanical Garden in Paris is reported to be Mexico (on sheet: “Mexique?”). This is a mistake since the species in endemic to New Caledonia (see also Hartley and Mabberley 2003).

**10.1. Melicopetrifoliatavar.trifoliata (Baill.) Appelhans & W.L.Wagner**

##### ﻿10.2. Melicope trifoliata var. gracilis

Taxon classificationPlantaeSapindalesRutaceae

(Baill.) Appelhans & W.L.Wagner comb. nov.

CABDE9C8-A20C-5678-8CE7-838BD6FB1A45

urn:lsid:ipni.org:names:77365270-1

Zieridium gracile Baill., Adansonia 10: 304. 1872.
Picrella trifoliata Baill. var. gracilis (Baill.) T.G.Hartley & Mabb., Adansonia sér. 3: 258. 2003.

###### Type material.

**New Caledonia**: 1861, *Deplanche 497* (lectotype, designated by Hartley and Mabberley 2003, pg. 256: P [P00222136]; isolectotype P [P00645790!], P-Baillon,); s.d., *Pancher 5996 p.p*. (syntype P [P00222137!]).

##### ﻿10.3. Melicope trifoliata var. gracillima

Taxon classificationPlantaeSapindalesRutaceae

(T.G.Hartley) Appelhans & W.L.Wagner comb. nov.

FAE387CB-ED8E-53A6-922C-CD0C0FA29AC8

urn:lsid:ipni.org:names:77365271-1

Picrella trifoliata Baill. var. gracillima T. G.Hartley, Adansonia sér. 3: 259. 2003.

###### Type material.

**New Caledonia**: Poya, Avangui, 11 Apr. 1969, *MacKee 20523* (holotype P [P00543932!], Isotype NOU [NOU001085!]).

#### ﻿Melicopesect.Sarcomelicope (Baill.) Appelhans & W.L.Wagner, stat. et comb. nov.

*Sarcomelicope* Engl., in Engler & Prantl, Nat. Pflanzenfamilien III, 4: 122. 1896.

*Bauerella* Borzi, Bol. Orto Bot. Palermo 1: 155. 1897.

**Type species**: *Melicopenervulosa* Pillon & Appelhans, nom. nov.

**Note.** Nine species, one of which is subdivided into three subspecies; distributed from Eastern Australia to Fiji, all but one species endemic to New Caledonia.

##### ﻿1. Melicope argyrophylla

Taxon classificationPlantaeSapindalesRutaceae

(Guill.) Appelhans & W.L.Wagner comb. nov.

FD90F51E-51F1-5671-8B9F-66F9DAB3BE6F

urn:lsid:ipni.org:names:77365272-1

Sarcomelicope argyrophylla Guill., Bull. Mus. Hist. Nat. Paris 26: 260. 1920.

###### Type material.

**New Caledonia**: Forêt près de la Plaine des Lacs, 15 Oct. 1914, *Franc 1895* (holotype P [P00543930!]; isotypes A [A00105549!, A00105550!, A00105551!], G [G00380810!, G00380811!], P [P00543928!, P00543929!, P00543930!]).

##### ﻿2. Melicope baueri

Taxon classificationPlantaeSapindalesRutaceae

(Schott) Appelhans & W.L.Wagner comb. nov.

6F9521C7-1D40-56D8-8675-D896020965CA

urn:lsid:ipni.org:names:77365273-1

Vepris simplicifolia Endl., Prod. Fl. Norfolk: 89. 1833.
Acronychia baueri Schott Fragm. Bot. 5. t. 3. 1834. Type: locality unknown, possibly Norfolk Island, Bauer s.n. (not located).
Acronychia simplicifolia (Endl.) Steud., Nomencl. Bot. [Steudel] 2, ed. 2: 747. 1841.
Acronychia hillii F.Muell., Fragm. (Mueller) 1: 26. 1858. Type: Australia, Queensland, Brisbane River, s.d., Hill s.n. (lectotype, designated here, MEL [MEL48088!]); Australia, Queensland, Brisbane River, Moreton Bay, Jul. 1855, von Mueller s.n. (syntypes BM [BM000798425!], K [K000717524!], MEL [MEL48077!]).
Jambolifera baueri (Schott) Kuntze, Revis. Gen. Pl. 1: 102. 1891. Type: Based on Acronychiabaueri Schott
Acronychia baueri Schott forma majoriflora Domin, Biblioth. Bot. 89: 294. 1927. Type: Australia, Queensland, Brisbane River, 1863–1865. Dietrich s.n. (holotype PR [PR 0528155!]; isotypes BRI [BRI-AQ0150877!], HBG!).
Bauerella baueri (Schott) Engl. ex Däniker, Vierteljahrsschr. Naturf. Ges. Zürich 77 (Beibl. 19): 202. 1932. Type: Based on Acronychiabaueri Schott
Acronychia simplicifolia (Endl.) McGill. & P.S.Green, J. Arnold Arb. 51: 209. 1970, isonym.
Acronychia simplicifolia (Endl.) Steudel ssp. simplicifolia , J. Arnold Arb. 51: 209, fig. 1a. 1970, as Acronychiasimplicifolia (Endl.) McGill. & P.S.Green ssp. simplicifolia.
Bauerella simplicifolia (Endl.) T.G.Hartley, J. Arnold Arb. 56: 168. 1975.
Bauerella simplicifolia (Endl.) T.G.Hartley ssp. simplicifolia , J. Arnold Arb. 56: 168, fig. 1b & 1c. 1975.
Sarcomelicope simplicifolia (Endl.) T.G.Hartley, Austral. J. Bot. 30: 369. 1982.
Sarcomelicope simplicifolia (Endl.) T.G.Hartley ssp. simplicifolia , Austral. J. Bot. 30: 370. 1982.

###### Type material.

**Australia**: Norfolk Island, s.d., *Bauer s.n.* (holotype W [W0046195!]; isotype W [W0046196!]).

###### Note.

The specific epithet *simplicifolia* is pre-empted in *Melicope*. *Melicopesimplicifolia* Domin is a synonym of *Melicopebroadbentiana* F.M.Bailey. The second oldest epithet “*baueri*” is used for this species accordingly.

**2.1. Melicopebauerisubsp.baueri**

##### ﻿2.2. Melicope baueri subsp. neo-scotica

Taxon classificationPlantaeSapindalesRutaceae

(P.S.Green) Appelhans & W.L.Wagner comb. nov.

05B2AA12-B7D2-530A-A6A6-A0FC1970CF37

urn:lsid:ipni.org:names:77365274-1

Acronychia simplicifolia (Endl.) Steud. subsp. neo-scotica P.S.Green, J. Arnold Arb. 51: 211, fig. 1b. 1970.
Bauerella simplicifolia (Endl.) T.G.Hartley ssp. neo-scotica (P.S.Green) T.G.Hartley, J. Arnold Arb. 56: 169. 1975.
Sarcomelicope simplicifolia (Endl.) T.G.Hartley ssp. neo-scotica (P.S.Green) T.G.Hartley, Austral. J. Bot. 30: 371. 1982.

###### Type material.

**New Caledonia**: Port Boisé, 1861–1867, *Deplanche 511* (holotype K [K000717519!]; isotypes P [P00543913!, P00543914!]).

##### ﻿2.3. Melicope baueri subsp. petiolaris

Taxon classificationPlantaeSapindalesRutaceae

(A.Gray) Appelhans & W.L.Wagner comb. nov.

EA4AD190-A371-587E-BAAD-74EBACF976C3

urn:lsid:ipni.org:names:77365275-1

Acronychia petiolaris A. Gray, U.S. Expl. Exped., Phan. 15: 335. I. 33. 1854.
Jambolifera petiolaris (A.Gray) Kuntze, Revis. Gen. Pl. 1: 102. 1891.
Acronychia simplicifolia (Endl.) Steudel ssp. petiolaris (A.Gray) P.S.Green J. Arnold Arb. 51: 212, fig. 1c. 1970.
Bauerella simplicifolia (Endl.) T.G.Hartley ssp. petiolaris (A.Gray) T.G.Hartley, J. Arnold Arb. 56: 169. 1975.
Bauerella petiolaris (A.Gray) A.C.Sm., Allertonia 1: 410. 1978.
Sarcomelicope simplicifolia (Endl.) T.G.Hartley ssp. petiolaris (A.Gray) T.G.Hartley, Austral. J. Bot. 30: 371. 1982.

###### Type material.

**FIJI**: Muthuata, 1838–1842, *U.S. Expl. Exped. s.n*. (holotype US [US00101682!]; isotype GH [GH00043963!]).

##### ﻿3. Melicope dogniensis

Taxon classificationPlantaeSapindalesRutaceae

(T.G.Hartley) Appelhans & W.L.Wagner comb. nov.

BB2628DA-E4E9-557A-B00E-AB2C89AE5F89

urn:lsid:ipni.org:names:77365276-1

Sarcomelicope dogniensis T. G.Hartley, Austral. J. Bot. 30: 367. 1982.

###### Type material.

**New Caledonia**: Plateau de Dogny, pente ouest, 30 Mar. 1965, *MacKee 12328* (holotype P [P00543927!]; isotypes K [K000717522!], P [P00543925!, P00543926!]).

##### ﻿4. Melicope follicularis

Taxon classificationPlantaeSapindalesRutaceae

(T.G.Hartley) Appelhans & W.L.Wagner comb. nov.

852F2326-7D40-5420-BC1C-9880DA278C32

urn:lsid:ipni.org:names:77365277-1

Sarcomelicope follicularis T. G.Hartley, Bull. Mus. Natl. Hist. Nat., B, Adansonia Sér. 4, 8(2): 183. 1986.

###### Type material.

**New Caledonia**: Pouébo, crête entre Mandjélia et Col de Tiébo, 19. Sep. 1973, *MacKee 27383* (holotype P [P00543924!]; isotypes NOU [NOU006501!], P [P00543922!, P00543923!]).

##### ﻿5. Melicope glauca

Taxon classificationPlantaeSapindalesRutaceae

(T.G.Hartley) Appelhans & W.L.Wagner comb. nov.

3485C945-B901-5F9B-892C-160A20F5B39E

urn:lsid:ipni.org:names:77365278-1

Sarcomelicope glauca T. G.Hartley, Austral. J. Bot. 30: 366. 1982.

###### Type material.

**New Caledonia**: Isle of Pines, Base du Pic Meunié près de la prise d`eau alimentant Kuto, 1 Mar. 1943, *Virot 1060* (holotype P [P00543921!]; isotype NOU [NOU006502!]).

##### ﻿6. Melicope leiocarpa

Taxon classificationPlantaeSapindalesRutaceae

(P.S.Green) Appelhans & W.L.Wagner comb. nov.

36C84302-45DB-5C7F-AC7A-0D789D8A2362

urn:lsid:ipni.org:names:77365279-1

Acronychia leiocarpa P.S.Green, J. Arnold Arbor. 51: 213. 1970.
Bauerella leiocarpa (P.S.Green) T.G.Hartley, J. Arnold Arbor. 56: 169. 1975.
Sarcomelicope leiocarpa (P.S.Green) T.G.Hartley, Austral. J. Bot. 30: 368. 1982.

###### Type material.

**New Caledonia**: North East slope of Ouen Toro, Nouméa, 26 Sep. 1963, *Green 1211* (holotype K [K000717518!]; isotypes A [A02289082!], NOU [NOU006503!], P [P00543920!]).

##### ﻿7. Melicope megistophylla

Taxon classificationPlantaeSapindalesRutaceae

(T.G.Hartley) Appelhans & W.L.Wagner comb. nov.

10F7E9C9-02C9-5849-853E-5B3A31582248

urn:lsid:ipni.org:names:77365280-1

Sarcomelicope megistophylla T. G.Hartley, Bull. Mus. Natl. Hist. Nat., B, Adansonia Sér 4, 8(2): 185. 1986.

###### Type material.

**New Caledonia**: Along access road to dam on Néaoua River, S of Houaliou, 7 May 1984, *McPherson 6524* (holotype CANB [CANB 350168.1]; isotypes MO [MO-2965994!], NOU [NOU006504!], P [P00543919!]).

##### ﻿8. Melicope pembaiensis

Taxon classificationPlantaeSapindalesRutaceae

(T.G.Hartley) Appelhans & W.L.Wagner comb. nov.

BBEEEB88-A2C7-59C3-BD2A-D26EFF5F6F10

urn:lsid:ipni.org:names:77365281-1

Sarcomelicope pembaiensis T. G.Hartley, Bull. Mus. Natl. Hist. Nat., B, Adansonia Sér 4, 8(2): 185. 1986.

###### Type material.

**New Caledonia**: Col d’Amieu, Mont Pembai, 8 Oct. 1984, *MacKee 42316* (leg. Pusset) (holotype CANB [CANB354214.1!]; isotypes NOU [NOU006505!], P [P00543918!]).

##### ﻿9. Melicope nervulosa

Taxon classificationPlantaeSapindalesRutaceae

Pillon & Appelhans nom. nov.

8A0986BF-E7E4-5C9D-A9FA-21E1C316ECBA

urn:lsid:ipni.org:names:77365282-1

Euodia sarcococca Baill., Adansonia 11: 301. 1875, as Evodia (Melicope) sarcococca.
Sarcomelicope sarcococca (Baill.) Engl., in Engler and Prantl, Nat. Pflanzenfam. III. iv.: 122. 1896.

###### Type material.

**New Caledonia**: Au Nord de la Conception, Feb. 1870, *Balansa 2797* (holotype P [P00543917!]; isotypes K [K000717520!, K000717521!], P [P00543915!, P00543916!]).

###### Note.

The specific epithet *sarcococca* is pre-empted in *Melicope*. *Melicopesarcococca* Lauterb. is currently treated as a synonym of *Melicopedurifolia* (K.Schum.) T.G.Hartley. The epithet *nervulosa* refers to the finely reticulated leaf veination of this species.

### ﻿Insertae sedis

#### ﻿Excluded species from Melicopesect.Melicope

These species are more closely related to sect. Pelea and further morphological studies are needed in order to evaluate if they should be united with sect. Pelea or if new sections within *Melicope* need to be established to accommodate them. Most species are distributed in New Guinea, five species are endemic to Fiji, two occur in NE Australia, one on the Solomon Islands and Vanuatu, and a more northern group of species is distributed from SW India to Borneo and Hainan (Hartley 2001).

*Melicopeaequata* T.G.Hartley [New Guinea & Bismarck Archipelago], *M.broadbentiana* F.M.Bailey [NE Australia], *M.burttiana* B.C.Stone [Solomon Islands, Vanuatu], *M.carrii* T.G.Hartley [New Guinea], *M.contermina* C.Moore & F.Muell. [Lord How Island], *M.dicksoniana* T.G.Hartley [New Guinea], *M.evansensis* (A.C.Sm.) A.C.Sm. [Fiji], *M.flaviflora* A.C.Sm. [Fiji], *M.goilalensis* T.G.Hartley [New Guinea], *M.homoeophylla* A.C.Sm. [Fiji], *M.indica* Wight [SW India], *M.jugosa* T.G.Hartley [Borneo], *M.longior* T.G.Hartley [Bismarck Archipelago], *M.macgregorii* T.G.Hartley [New Guinea], *M.mucronata* Merr. & L.M.Perry [New Guinea], *M.novoguineensis* Valeton [New Guinea], *M.oblanceolata* T.G.Hartley [New Guinea], *M.patulinervia* (Merr. & Chun) C.C.Huang [Hainan, China], *M.perryae* T.G.Hartley [New Guinea], *M.petiolaris* T.G.Hartley [New Guinea], *M.phanerophlebia* (Merr. & L.M.Perry) T.G.Hartley [New Guinea], *M.pubifolia* Merr. & L.M.Perry [New Guinea], *M.reticulata* Lauterb. [New Guinea], *M.ridsdalei* T.G.Hartley [New Guinea], *M.robusta* A.C.Sm. [Fiji], *M.sororia* T.G.Hartley [Borneo], *M.stellulata* T.G.Hartley [New Guinea], *M.suberosa* Β.C.Stone [Malaysia, Peninsula], *M.sudestica* T.G.Hartley [Sudest Island, New Guinea], *M.taveuniensis* A.C.Sm. [Fiji], *M.trachycarpa* Lauterb. [New Guinea], *M.woitapensis* T.G.Hartley [New Guinea], *M.xanthoxyloides* (F.Muell.) T.G.Hartley [New Guinea, Bismarck Archipelago, NE Australia].

#### ﻿Taxa excluded from *Melicope*

***Euodiavitiflora* F.Muell., Fragm. 7: 144. 1871.**

*Melicopevitiflora* (F.Muell.) T.G.Hartley, Telopea 4: 34. 1990.

**Type material. Australia**: Queensland, North Kennedy, Rockingham Bay, 19 Oct. 1870, *Dallachy s.n.* (holotype MEL [MEL67570!]; isotype MEL [MEL64768!]).

**Note.** Excluding *M.vitiflora* renders *Melicope* monophyletic. The relationships of *M.vitiflora* are not fully clear. It is most closely related to the monotypic *Pitaviaster* T.G.Hartley and *Euodiapubifolia* T.G.Hartley (Appelhans et al. 2014b). We propose to treat it as a species of *Euodia* until its relationships are better understood.

## Supplementary Material

XML Treatment for Melicope fulva

XML Treatment for Melicope glaberrima

XML Treatment for Melicope gordonii

XML Treatment for Melicope lasioneura

XML Treatment for Melicope pedicellata

XML Treatment for Melicope vieillardii

XML Treatment for Melicope amosensis

XML Treatment for Melicope baudouinii

XML Treatment for Melicope drupacea

XML Treatment for Melicope fruticosa

XML Treatment for Melicope hartleyi

XML Treatment for Melicope homedeboense

XML Treatment for Melicope lactea

XML Treatment for Melicope lactea var. poissonii

XML Treatment for Melicope microcarpa

XML Treatment for Melicope oreophila

XML Treatment for Melicope oreophila var. longipes

XML Treatment for Melicope trifoliolata

XML Treatment for Melicope balgooyi

XML Treatment for Melicope lucida

XML Treatment for Melicope mantellii

XML Treatment for Melicope simplex

XML Treatment for Melicope tahitensis

XML Treatment for Melicope ternata

XML Treatment for Melicope bracteata

XML Treatment for Melicope capillacea

XML Treatment for Melicope erromangensis

XML Treatment for Melicope glandulosa

XML Treatment for Melicope ignambiensis

XML Treatment for Melicope laevis

XML Treatment for Melicope littoralis

XML Treatment for Melicope margaretae

XML Treatment for Melicope polybotrya

XML Treatment for Melicope trifoliata

XML Treatment for Melicope trifoliata var. gracilis

XML Treatment for Melicope trifoliata var. gracillima

XML Treatment for Melicope argyrophylla

XML Treatment for Melicope baueri

XML Treatment for Melicope baueri subsp. neo-scotica

XML Treatment for Melicope baueri subsp. petiolaris

XML Treatment for Melicope dogniensis

XML Treatment for Melicope follicularis

XML Treatment for Melicope glauca

XML Treatment for Melicope leiocarpa

XML Treatment for Melicope megistophylla

XML Treatment for Melicope pembaiensis

XML Treatment for Melicope nervulosa

## ﻿Additional information

### Author ORCIDs

Marc S. Appelhans https://orcid.org/0000-0003-4864-5003

Yohan Pillon https://orcid.org/0000-0003-1760-329X

Karine Gotty https://orcid.org/0000-0002-1465-5644

Warren L. Wagner https://orcid.org/0000-0001-5012-8422

### Data availability

All of the data that support the findings of this study are available in the main text.
